# Long-term oncologic outcomes after non-intubated versus intubated video-assisted thoracoscopic surgery for non-small cell lung cancer: a systematic review and meta-analysis

**DOI:** 10.3389/fonc.2026.1933004

**Published:** 2026-08-14

**Authors:** Yin-sheng Liao, Yu-ping Deng, Jian-li Song, Xuan Yu, Qiang Li, Bin Lu, Guan-yu Chen

**Affiliations:** 1 Department of Anesthesiology, Zigong Fourth People’s Hospital, Zigong, Sichuan, China; 2 Department of Anesthesiology, Zigong Hospital of Chengdu University of Traditional Chinese Medicine, Zigong, Sichuan, China

**Keywords:** disease-free survival, meta-analysis, non-intubated thoracic surgery, non-small cell lung cancer, overall survival, spontaneous ventilation, video-assisted thoracoscopic surgery

## Abstract

**Background:**

Non-intubated video-assisted thoracoscopic surgery (NIVATS) is increasingly used for selected non-small cell lung cancer (NSCLC) patients, but its long-term oncologic outcomes versus conventional VATS remain unclear. We therefore conducted this systematic review and meta-analysis to compare long-term oncologic outcomes between NIVATS and conventional intubated video-assisted thoracoscopic surgery (VATS).

**Methods:**

We searched major databases and trial registries through June 30, 2026 for comparative studies evaluating NIVATS or spontaneous ventilation VATS versus intubated or mechanical ventilation VATS in NSCLC. The primary outcomes were overall survival (OS) and disease-free survival or recurrence-free survival (DFS/RFS). Secondary oncologic outcomes included recurrence, R0 resection, total lymph node assessment, N1 and N2 lymph node assessment, and lymph node station or group assessment. Hazard ratios (HRs), risk ratios (RRs), and mean differences (MDs) with their 95% confidence intervals (CIs) were pooled using random-effects models. Risk of bias was assessed using the Risk of Bias 2 (RoB 2) tool and the Newcastle-Ottawa Scale (NOS), and the certainty of evidence was evaluated using the GRADE framework.

**Results:**

Seventeen comparative studies were included in the qualitative synthesis. NIVATS was associated with lower pooled hazards for OS compared with intubated VATS (HR 0.66, 95% CI 0.48-0.91; I^2^ = 0.0%; GRADE certainty: low) and lower pooled hazards for DFS/RFS (HR 0.66, 95% CI 0.50-0.87; I^2^ = 0.0%; GRADE certainty: low). These findings remained stable in sensitivity analyses. No significant difference was observed in total lymph nodes harvested or R0 resection rate. Recurrence showed a borderline lower risk after NIVATS, although follow-up definitions varied across studies. The randomized study was judged as having some concerns, and observational studies were generally moderate to high quality.

**Conclusions:**

In selected NSCLC patients, NIVATS does not appear to compromise long-term oncologic outcomes versus conventional intubated VATS. The observed survival advantage likely reflects selection bias and residual confounding, as NIVATS is often used in patients with favorable tumor features, operative suitability, or cardiopulmonary reserve. These findings support NIVATS as feasible under strict selection by experienced teams. Prospective multicenter studies with standardized protocols and long-term follow-up are needed to confirm its oncologic safety.

**Systematic review registration:**

crd.york.ac.uk/prospero/display_record.php?ID=CRD420261437783, identifier CRD420261437783.

## Introduction

Video-assisted thoracoscopic surgery (VATS) has become an established approach for the surgical treatment of early-stage and selected locally advanced non-small cell lung cancer (NSCLC). Its value lies not only in reducing surgical trauma but also in preserving the oncologic principles that determine long-term outcome, including complete resection, adequate nodal evaluation, and durable disease control (1–5). As surgical techniques have matured, perioperative management has also evolved. One of the most notable developments is non-intubated VATS (NIVATS), which allows thoracoscopic lung resection under spontaneous ventilation without endotracheal intubation, neuromuscular blockade, or conventional one-lung mechanical ventilation (1–5).

The rationale for NIVATS is clinically and biologically plausible. Avoiding tracheal intubation and mechanical ventilation may reduce airway injury, ventilator-associated lung stress, residual neuromuscular effects, and anesthesia-related inflammatory disturbance (1–5). Several comparative studies have reported favorable perioperative findings after NIVATS, including shorter fasting time, less postoperative drainage, earlier recovery, shorter hospitalization, and improved preservation of immune or inflammatory markers (1–5). These potential benefits are particularly attractive for elderly patients, patients with impaired pulmonary function, overweight patients, and other populations in whom conventional general anesthesia may increase perioperative vulnerability.

However, perioperative benefit alone is not sufficient to justify a change in anesthetic strategy for cancer surgery. For NSCLC, the more important question is whether NIVATS can maintain long-term oncologic safety. Concerns remain that spontaneous ventilation, diaphragmatic movement, mediastinal shift, cough reflex, or insufficient operative exposure might affect the quality of lymph node dissection, margin status, or intraoperative decision-making. In addition, the selection of patients for NIVATS is often conservative, and many published studies are retrospective or single-center experiences. These features make it difficult to determine whether observed long-term outcomes reflect the anesthetic strategy itself, patient selection, institutional expertise, or differences in tumor biology.

Several previous systematic reviews and meta-analyses have evaluated non-intubated VATS, but most focused on feasibility, perioperative complications, short-term recovery, conversion, operative time, length of stay, or lymph-node yield rather than long-term survival (6–10). Existing studies have reported OS, DFS, RFS, recurrence, lymph node dissection, and margin outcomes, but the evidence has remained fragmented. Some studies suggest that NIVATS may be associated with better survival or fewer recurrences, whereas others report comparable long-term outcomes. Moreover, fixed-time survival rates, hazard ratios, recurrence events, and lymph node indicators have not always been reported consistently. Therefore, a synthesis focused specifically on long-term prognosis is needed. Such a synthesis should evaluate survival outcomes together with oncologic adequacy indicators, because any apparent survival advantage would be difficult to interpret if it were accompanied by inferior lymph node assessment or incomplete resection.

We conducted this systematic review and meta-analysis to compare long-term oncologic outcomes after NIVATS versus conventional intubated or mechanical ventilation VATS in patients with NSCLC. The primary endpoints were OS and DFS/RFS. Secondary endpoints included recurrence, R0 resection, total lymph node assessment, N1 and N2 lymph node assessment, and lymph node station or group assessment. Our aim was to determine whether NIVATS is associated with long-term outcomes comparable to those of intubated VATS while preserving the oncologic standards required for lung cancer surgery.

## Methods

### Study design and reporting

This systematic review and meta-analysis was conducted following the Preferred Reporting Items for Systematic Reviews and Meta-Analyses (PRISMA) 2020 statement (11). The protocol for this meta-analysis is available in PROSPERO (CRD420261437783).

### Search strategy

Two authors (YL, YD) independently searched PubMed, Web of Science, Cochrane Library, ClinicalTrials.gov, and Embase from inception to June 30, 2026 for comparative studies evaluating NIVATS or spontaneous ventilation VATS versus intubated or mechanical ventilation VATS in NSCLC. Search terms included combinations of terms related to non-intubated thoracic surgery, spontaneous ventilation, video-assisted thoracoscopic surgery, lung cancer, non-small cell lung cancer, survival, recurrence, and prognosis. The full database-specific search strategy is provided in **Supplementary File 1**. Reference lists of included studies and relevant systematic reviews were also screened to identify additional eligible studies. Disagreements between the two reviewers were resolved by discussion with a third author.

### Eligibility criteria

Eligibility criteria were defined according to the PICOS framework.

1. Population: adults with pathologically or clinically diagnosed NSCLC undergoing VATS, including studies with clearly extractable NSCLC subgroup data.
2. Intervention: NIVATS, non-intubated thoracoscopic surgery, awake VATS, tubeless VATS, or spontaneous-ventilation VATS.
3. Comparator: conventional intubated VATS under general anesthesia with mechanical or one-lung ventilation.
4. Outcomes: at least one long-term oncologic outcome or oncologic adequacy outcome, including OS, DFS/RFS, recurrence, death events, R0 resection, lymph node number, or lymph node station/group number.
5. Study design: randomized trials and comparative observational studies, including cohort studies and propensity score-matched analyses. Eligible studies had to provide sufficient data for quantitative synthesis or clinically meaningful narrative synthesis.
6. Language: full-text articles published in English.

Studies were excluded if they were reviews, editorials, case reports, conference abstracts without usable data, non-English articles, duplicate publications, or non-comparative series. Studies with mixed benign and malignant diseases, mixed thoracic indications, or lung cancer cohorts including non-NSCLC histologies were excluded from the main analysis unless NSCLC-specific data could be extracted. Such studies were retained only for narrative interpretation or sensitivity analysis when clinically relevant.

### Outcomes

The primary outcomes were OS and DFS/RFS. OS was defined as time from surgery to death from any cause. DFS/RFS was defined according to the definitions used in each included study and generally represented time to recurrence, disease progression, or death. Because individual studies used DFS and RFS inconsistently, these outcomes were combined as DFS/RFS for quantitative synthesis.

Secondary outcomes included total lymph nodes harvested or dissected, N1 lymph node number, N2 lymph node number, total lymph node station or group number, N1 and N2 station or group number, recurrence, death events, and R0 resection. When multiple lymph node metrics were available, node number and station/group number were analyzed separately to avoid combining distinct surgical quality indicators.

### Data extraction

Two reviewers independently extracted data using a standardized form (YL, YD). Extracted information included first author, publication year, country or region, study design, population characteristics, sample size, age, tumor stage, surgical procedure, anesthetic strategy, matching method, follow-up duration, and reported oncologic outcomes. For studies contributing to the primary survival analyses, we additionally extracted or recorded clinical and pathological stage distribution, radiologic GGO/solid phenotype, pure or predominant GGO proportion, tumor size or solid-component size, tumor biological or pathological factors, follow-up duration, and variables used for matching or multivariable adjustment when available. For survival outcomes, HRs and 95% confidence intervals (CIs) were preferentially extracted from the original text, tables, or figures. When HRs were not directly reported but survival estimates were available, reconstructed survival HRs were incorporated into the primary pooled analysis when appropriate, whereas fixed-time estimates were used only in exploratory fixed-time analyses; concurrently, sensitivity analyses (e.g., restricting to studies that directly reported HRs) were performed to assess the impact of these estimates on the pooled results and to test the robustness of the findings. Any discrepancies were resolved by discussion with a third reviewer (GC).

### Risk of bias assessment

Risk of bias was assessed independently by two reviewers (YL, YD). Randomized studies were evaluated using the Cochrane RoB 2 tool, which assesses bias arising from the randomization process, deviations from intended interventions, missing outcome data, outcome measurement, and selection of the reported result (12). Observational studies were assessed using the Newcastle-Ottawa Scale (NOS), covering selection of cohorts, comparability of groups, and outcome assessment (13). Certainty of evidence for major outcomes was graded using the GRADE framework, considering risk of bias, inconsistency, indirectness, imprecision, and publication bias (14). Disagreements were resolved by discussion and, when needed, adjudication by a third reviewer (GC).

### Statistical analysis

For time-to-event outcomes, hazard ratios (HRs) and 95% confidence intervals (CIs) were used as the primary effect measures. Multivariable Cox model estimates were preferentially extracted. When multivariable estimates were unavailable, univariable, figure-reported, abstract-reported, or directly reported HRs were used. The direction of effect was harmonized so that HRs below 1 favored non-intubated or spontaneous-ventilation VATS. HRs were transformed to the natural logarithmic scale before pooling using logHR = ln(HR). Standard errors were calculated from the reported 95% CIs using SE(logHR) = [ln(upper CI) - ln(lower CI)]/3.92, according to established methods for incorporating summary time-to-event outcomes into meta-analysis (15).

When HRs were not directly reported but Kaplan-Meier curves were available, curves were digitized from high-resolution figures. When sufficient information was available, including numbers at risk and follow-up time points, pseudo-individual patient data were reconstructed according to the algorithm described by Guyot et al. (16). Reconstructed survival data were analyzed using Cox proportional hazards models to obtain HRs and 95% CIs. These reconstructed estimates were classified according to source type and examined in sensitivity or exploratory analyses.

For exploratory fixed-time survival analyses, 1-, 3-, and 5-year survival probabilities were extracted from tables, text, or Kaplan-Meier curves when available. Cumulative hazards were estimated as H(t) = -ln[S(t)], and fixed-time HRs were approximated as HR(t) = H_non-intubated(t)/H_intubated(t). When no timepoint-specific standard error was available, the standard error was conservatively anchored using the reported full-follow-up HR or Cox-model CI from the same study. These estimates were classified as fixed-time survival estimates, KM-calibrated estimates, or digitized KM estimates and were used only for exploratory fixed-time analyses, not as replacements for the primary full-follow-up HR-based pooled analyses. If both arms had zero events at a fixed time point, the HR was considered not estimable and no arbitrary continuity correction was applied. Approximate event numbers from fixed-time survival rates were calculated descriptively as events ≈ n × [1 - S(t)] but were not used to replace time-to-event HRs when HR data were available.

For dichotomous outcomes, including recurrence, death, and R0 resection, risk ratios (RRs) and 95% CIs were calculated from extracted event counts and group totals. The standard error of logRR was calculated as SE(logRR) = sqrt(1/a - 1/n1 + 1/c - 1/n0), where a and c represent event counts in the non-intubated and intubated groups, respectively, and n1 and n0 represent the corresponding group sizes. R0 resection was directly extracted when reported; when only positive-margin events were reported, R0 events were derived as total patients minus positive-margin cases.

For continuous outcomes, including total lymph nodes, N1 lymph nodes, N2 lymph nodes, and lymph-node stations or groups, means, standard deviations, and sample sizes were extracted directly from full texts or tables. Mean differences (MDs) were calculated as MD = mean_non-intubated - mean_intubated, with standard errors calculated as SE(MD) = sqrt(SD1²/n1 + SD0²/n0). Median and interquartile range data were not converted for the final pooled analyses unless sufficient information was available and the outcome was judged clinically and statistically compatible with mean-based pooling.

Random-effects meta-analyses were conducted using the rma() function in the metafor package with restricted maximum likelihood (REML) estimation for between-study variance (17). Pooled estimates were reported with 95% CIs and two-sided P values. Statistical heterogeneity was evaluated using Cochran’s Q test, tau-squared, and I-squared statistics (18). Sensitivity analyses included core-study-only pooling and leave-one-out analyses. Subgroup analyses were performed only when each subgroup contained at least two studies. Meta-regression was used to explore whether age group, follow-up duration, sample size, surgical scope, or geographic region modified pooled OS or DFS/RFS estimates; because of the limited number of studies, these analyses were considered exploratory.

Small-study effects were assessed using funnel plots and Egger’s test when sufficient studies were available. Because fewer than 10 studies were available for the primary outcomes, publication-bias analyses were interpreted as exploratory (19). All forest plots, subgroup plots, sensitivity plots, fixed-time survival plots, funnel plots, and meta-regression plots were generated in R.

All statistical analyses and figure generation were performed by GC using R version 4.6.0 (R Foundation for Statistical Computing, Vienna, Austria) (20). The analytical codes and output were independently cross-checked by BL and JS to ensure the accuracy and reproducibility of the results. Data were imported using the readxl package, cleaned and reshaped with dplyr and tidyr, analyzed with metafor, exported with writexl, and visualized using ggplot2, grid, and gridExtra (21–26).

## Results

### Study selection and characteristics

**Figure 1:**
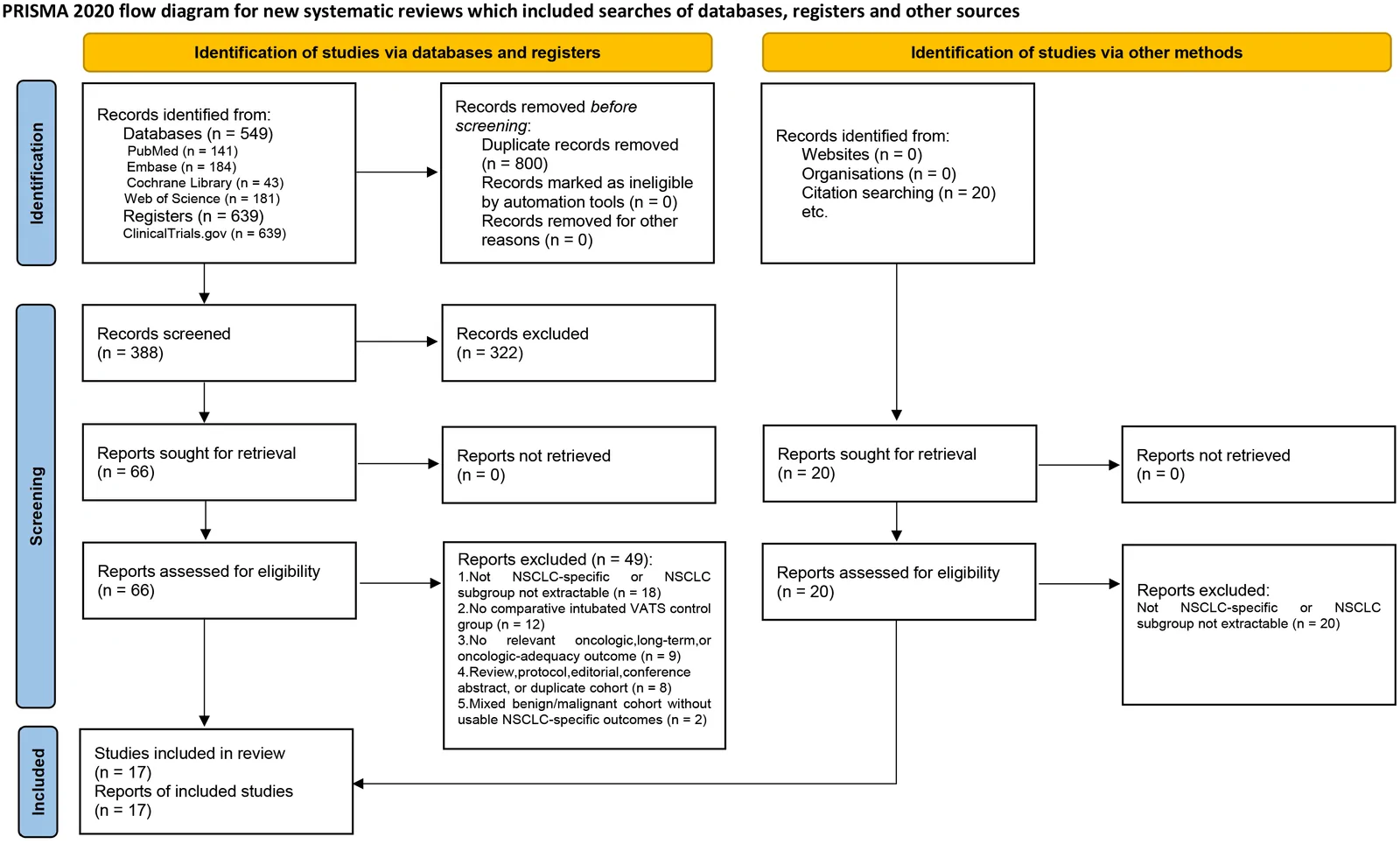
PRISMA flow diagram. Flow diagram showing record identification from databases, registers, and citation searching, duplicate removal, title and abstract screening, full-text eligibility assessment, reasons for exclusion, and final inclusion of studies in the systematic review and meta-analysis.

A total of 1, 188 records were identified from database and register searches, and 20 additional records through citation searching. After removing duplicates, 388 records remained for title/abstract screening, of which 322 were excluded. The remaining 66 full-text articles and the 20 citation-searched records were assessed. Of the 66 database/register reports, 49 were excluded: 18 not NSCLC-specific, 12 no intubated control group, 9 no relevant oncologic outcomes, 8 review/protocol/editorial/abstract/duplicate, and 2 mixed benign/malignant cohort. All 20 citation-searched records were excluded for not meeting eligibility criteria. Ultimately, 17 studies were included. The screening process is shown in **Figure 1**. All supplementary tables are included within **Supplementary File 2**, and the completed PRISMA 2020 checklist is provided as **Supplementary File 3**.

**Supplementary Table 1** summarizes the characteristics of the 17 included studies, including study design, eligibility criteria, tumor/NSCLC-extractable sample size, anesthetic technique, surgical procedure, tumor stage/profile, and extractable oncologic outcomes. The tumor/NSCLC-extractable sample size was 2250 patients, counting only NSCLC or clearly extractable target lung-cancer oncologic data and excluding benign or non-target thoracic cases from the denominator. The included studies were published between 2011 and 2026 and represented cohorts from China, South Korea, Hungary, Thailand, and the United States. Most studies were retrospective comparative cohorts, frequently using propensity score matching or weighting; one randomized clinical study was included. Study populations included clinical stage I NSCLC, invasive stage I-III NSCLC, elderly and octogenarian NSCLC patients, patients with poor pulmonary function, overweight or obese patients, and patients receiving neoadjuvant or adjuvant therapy (27–43).

The surgical procedures included VATS lobectomy, segmentectomy, wedge resection, anatomical resection, and mixed thoracoscopic lung resections. The non-intubated/spontaneous-ventilation strategies varied across studies and included laryngeal-mask-assisted spontaneous ventilation, deep sedation with spontaneous breathing without tracheal intubation, thoracic epidural or regional/local anesthesia with sedation, intravenous anesthesia with spontaneous ventilation, and combined non-intubated anesthetic approaches. Control groups consistently underwent conventional intubated general anesthesia with one-lung mechanical ventilation. Not all studies reported long-term survival outcomes; some contributed only to secondary oncologic adequacy outcomes, particularly lymph node assessment, R0 resection, recurrence events, or adjuvant chemotherapy delivery.

Three included reports were not strictly pure NSCLC cohorts at the article level and were therefore handled cautiously. Guo et al. included benign and malignant segmentectomy cases, Yu et al. included a lung cancer cohort with rare non-NSCLC histologies, and Udelsman et al. included mixed thoracoscopic indications (30, 40, 42). These studies were retained only when NSCLC-specific or clearly extractable target lung-cancer oncologic data could be isolated; benign cases, non-oncologic thoracic indications, and non-target histologies were not counted in the tumor/NSCLC-extractable total. Studies without extractable target-population survival estimates were not included in the corresponding quantitative survival meta-analysis.

For the studies contributing to OS or DFS/RFS analyses, **Supplementary Table 2** further summarizes baseline tumor and comparability features relevant to potential selection bias, including clinical and pathological stage distribution, radiologic GGO/solid phenotype, pure or predominant GGO proportion, tumor size or solid-component size, tumor biological/pathological factors, follow-up duration, and matching or adjustment variables. Clinical or pathological stage information was available in all survival-contributing studies, and most cohorts were predominantly early-stage or stage I-enriched. However, pure/predominant GGO proportion, solid tumor proportion, solid-component size, and detailed molecular or biological tumor features were incompletely and inconsistently reported. These unreported variables were recorded as NR rather than assumed to be balanced.

### Risk of bias and certainty of evidence

One randomized clinical study was assessed using RoB 2 and was judged as having some concerns overall, mainly because of concerns related to the randomization process, deviations from intended interventions, outcome measurement, and selection of the reported result. The observational studies were assessed using the Newcastle-Ottawa Scale; most were rated as moderate or moderate-to-high quality, with lower scores mainly reflecting retrospective design, residual confounding, and limitations in follow-up or outcome assessment (**Supplementary Table 3** and **S4**). According to GRADE, the certainty of evidence was low for OS and DFS/RFS, mainly because the evidence was derived predominantly from non-randomized studies and because of imprecision and endpoint heterogeneity. Certainty was low or very low for most secondary outcomes, particularly outcomes based on sparse events or few studies (**Supplementary Table 5**). These GRADE findings were used to temper the interpretation of the pooled survival estimates and to avoid causal claims based on predominantly observational evidence.

### Overall survival and disease-free or recurrence-free survival

**Figure 2:**
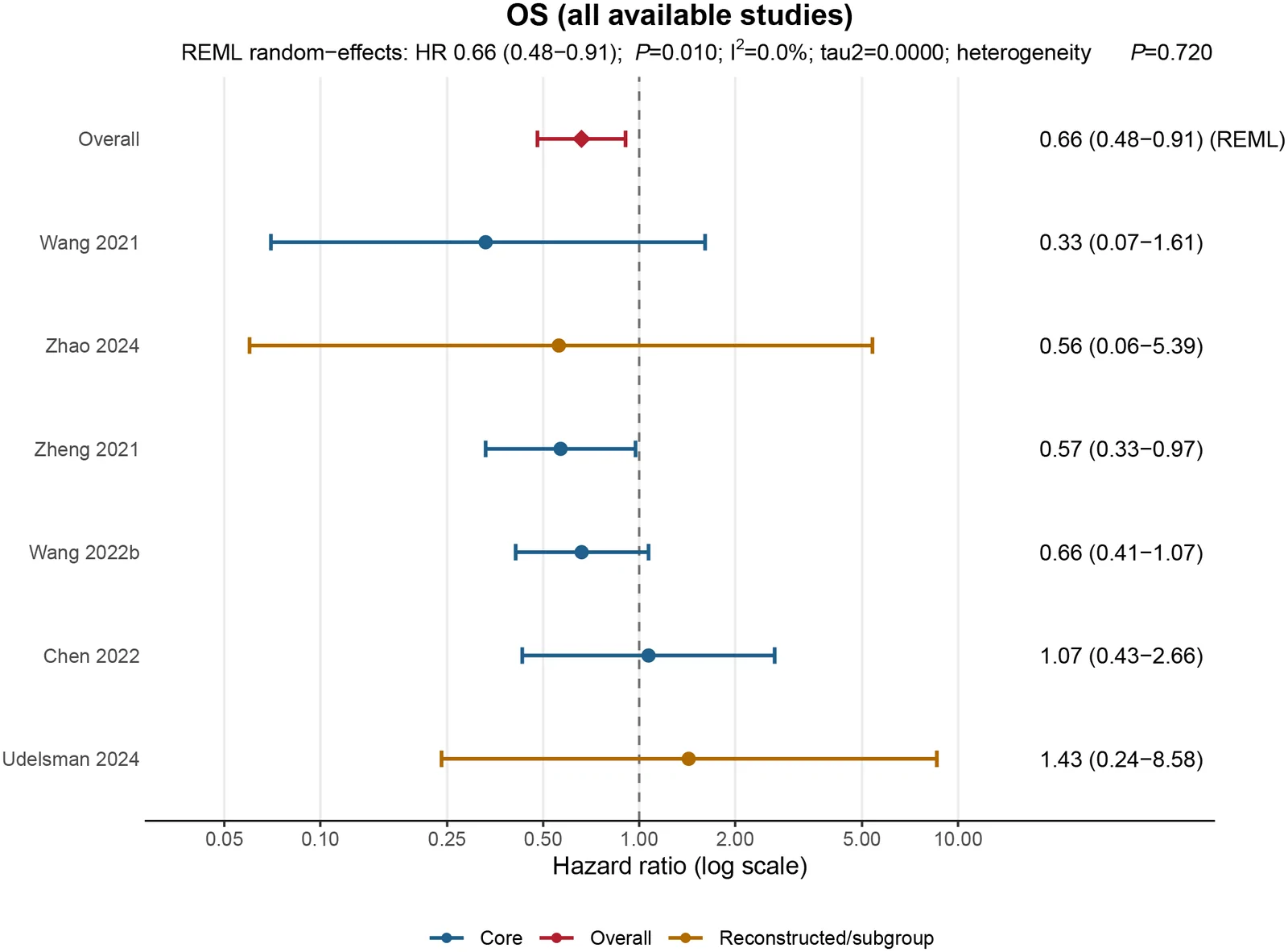
Overall survival after non-intubated/spontaneous-ventilation VATS versus intubated VATS. Forest plot of hazard ratios for overall survival using a random-effects restricted maximum likelihood model. Hazard ratios below 1 favor non-intubated/spontaneous-ventilation VATS. The pooled estimate was HR 0.66 (95% CI 0.48-0.91; P = 0.010; I² = 0.0%).

Six studies contributed to the primary pooled OS analysis. Compared with intubated VATS, NIVATS was associated with lower pooled hazards for OS (HR 0.66, 95% CI 0.48-0.91; P = 0.010). No statistical heterogeneity was observed (I² = 0.0%; heterogeneity P = 0.720) (**Figure 2**).

**Figure 3:**
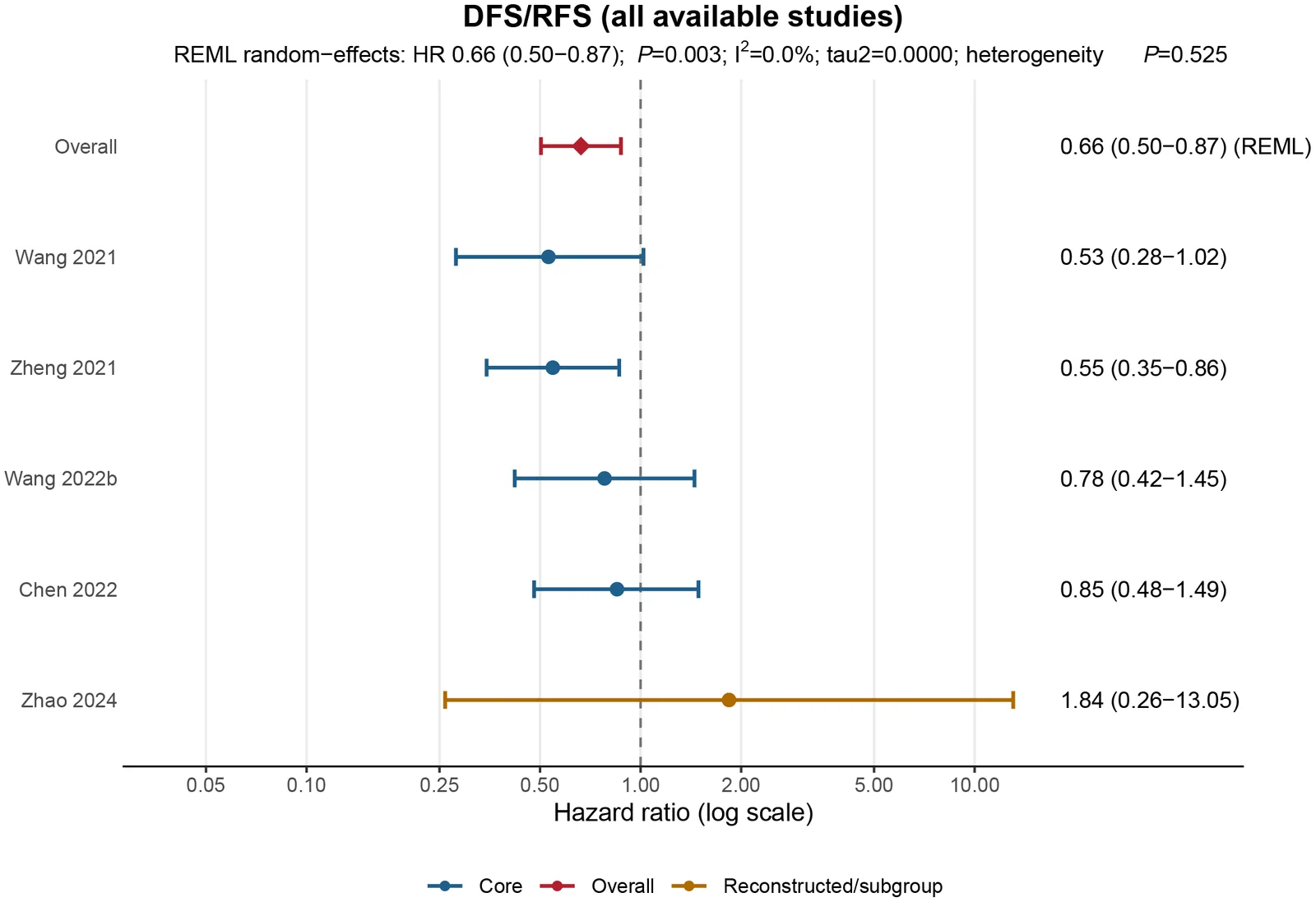
Disease-free or recurrence-free survival after non-intubated/spontaneous-ventilation VATS versus intubated VATS. Forest plot of hazard ratios for DFS/RFS using a random-effects restricted maximum likelihood model. Hazard ratios below 1 favor non-intubated/spontaneous-ventilation VATS. The pooled estimate was HR 0.66 (95% CI 0.50-0.87; P = 0.003; I² = 0.0%).

Five studies contributed to the primary pooled DFS/RFS analysis. NIVATS was associated with lower pooled hazards for DFS/RFS compared with intubated VATS (HR 0.66, 95% CI 0.50-0.87; P = 0.003), with no observed heterogeneity (I^2^ = 0.0%; heterogeneity P = 0.525) (**Figure 3**).

The direction of the DFS/RFS result was consistent with the OS analysis. However, definitions of DFS and RFS varied across studies, and not all studies distinguished recurrence from death as a component of the endpoint. Therefore, the pooled DFS/RFS estimate should be interpreted as a composite recurrence-related survival endpoint rather than a uniform endpoint across all studies.

### Time-specific survival analyses

**Figure 4:**
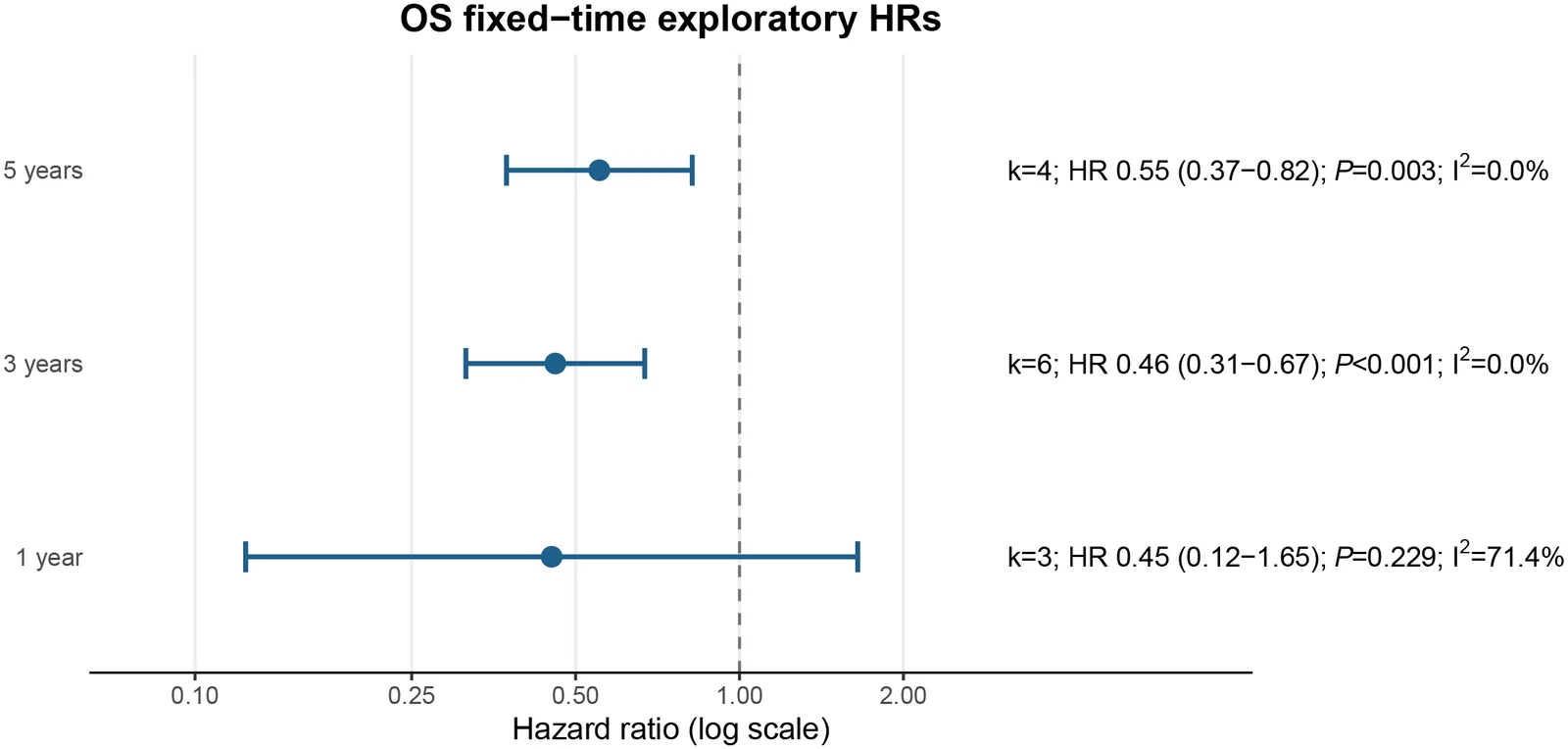
Exploratory fixed-time overall survival analyses. Summary hazard ratios for 1-year, 3-year, and 5-year OS based on available fixed-time survival estimates, Kaplan-Meier readings, or reconstructed estimates. These analyses were exploratory and were not used to replace the primary full-follow-up HR analysis.

**Figure 5:**
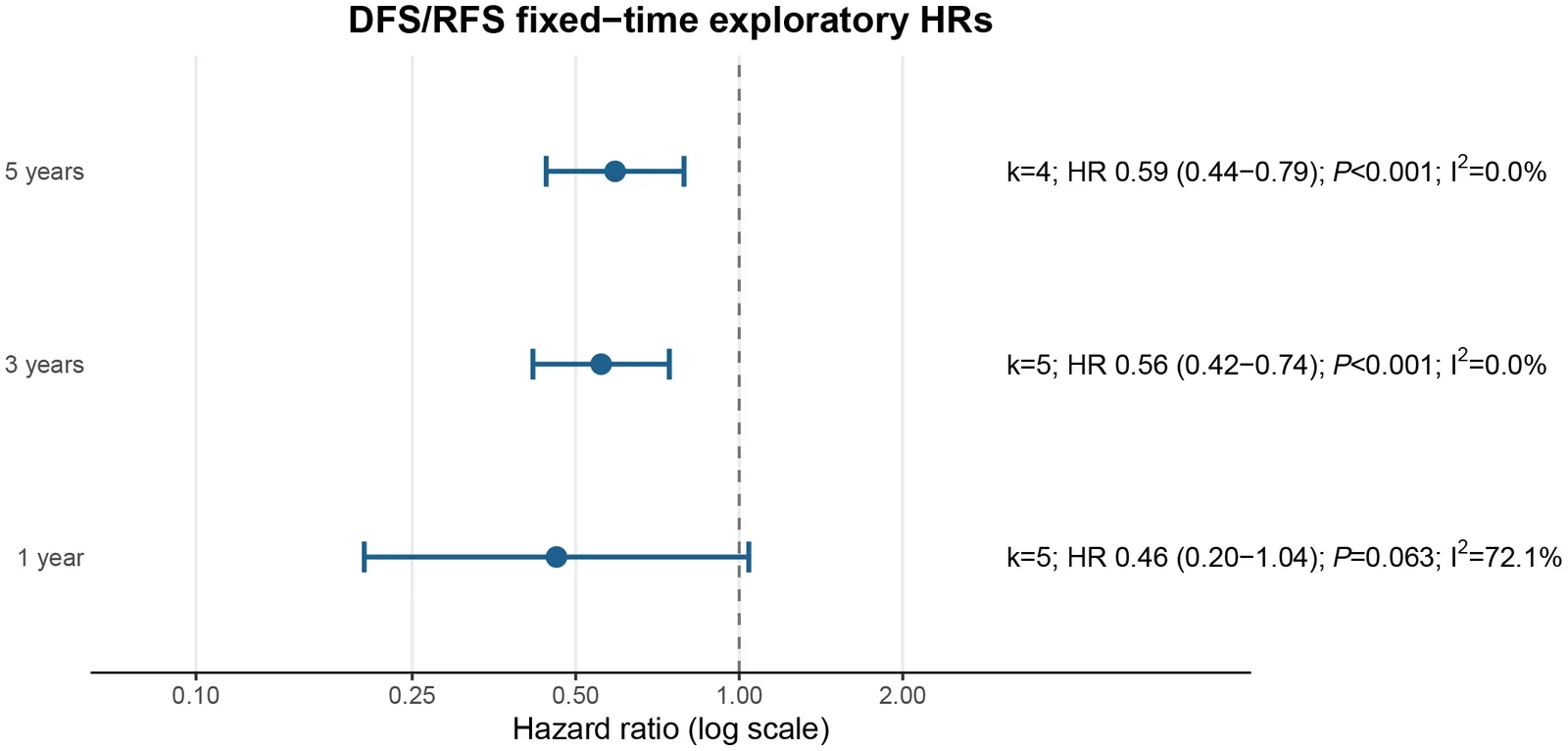
Exploratory fixed-time DFS/RFS analyses. Summary hazard ratios for 1-year, 3-year, and 5-year DFS/RFS based on available fixed-time survival estimates, Kaplan-Meier readings, or reconstructed estimates. These analyses were exploratory and were not used to replace the primary full-follow-up HR analysis.

Exploratory fixed-time analyses were performed for 1-year, 3-year, and 5-year survival estimates when data were available. For OS, the pooled estimate was imprecise at 1 year (HR 0.45, 95% CI 0.12-1.65), but favored NIVATS at 3 years (HR 0.46, 95% CI 0.31-0.67) and 5 years (HR 0.55, 95% CI 0.37-0.82) (**Figure 4**). For DFS/RFS, the 1-year estimate was also imprecise (HR 0.46, 95% CI 0.20-1.04), whereas 3-year and 5-year estimates favored NIVATS (3-year HR 0.56, 95% CI 0.42-0.74; 5-year HR 0.59, 95% CI 0.44-0.79) (**Figure 5**).

These time-specific analyses were considered exploratory because some estimates were derived from fixed-time survival rates, Kaplan-Meier readings, or reconstructed data rather than directly reported HRs. They were therefore used to examine consistency across follow-up intervals rather than to replace the primary HR-based analyses.

### Lymph node assessment and resection radicality

**Figure 6:**
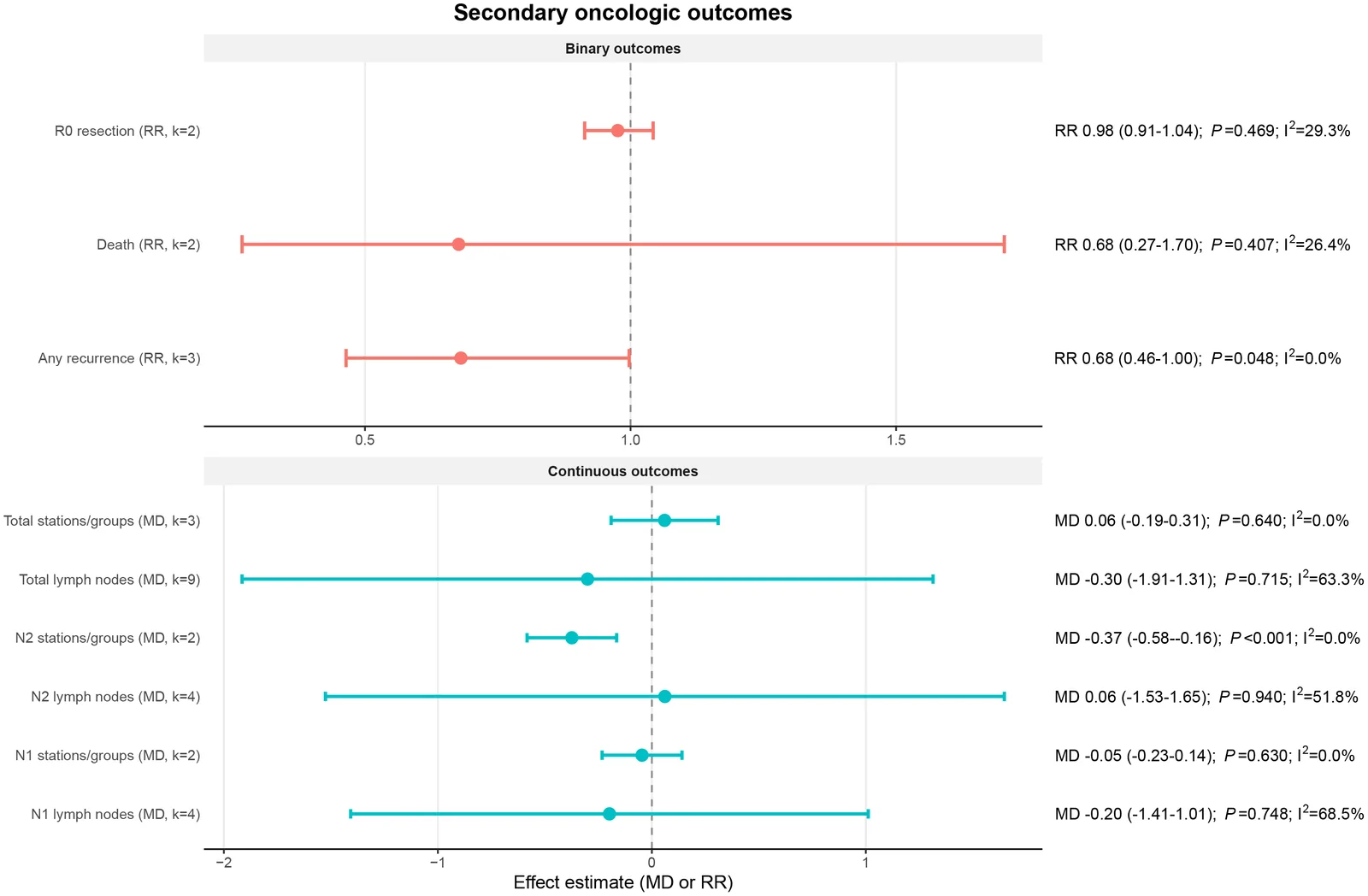
Secondary oncologic outcomes. Pooled analyses of recurrence, death, R0 resection, lymph-node yield, and lymph-node station/group assessment. Binary outcomes are summarized as risk ratios and continuous outcomes as mean differences. Values below 1 for risk ratios favor non-intubated/spontaneous-ventilation VATS when the event is adverse.

Secondary analyses were performed to determine whether the survival findings were accompanied by any evidence of compromised oncologic surgery (**Figure 6**). Total lymph nodes harvested did not differ significantly between NIVATS and intubated VATS (MD -0.30, 95% CI -1.91 to 1.31; I² = 63%). Similarly, N1 lymph node number (MD -0.20, 95% CI -1.41 to 1.01) and N2 lymph node number (MD 0.06, 95% CI -1.53 to 1.65) showed no significant between-group differences.

For lymph node station or group assessment, total station/group number was similar between groups (MD 0.06, 95% CI -0.19 to 0.31). N1 station/group number also did not differ significantly (MD -0.05, 95% CI -0.23 to 0.14). However, N2 station/group number was lower in the NIVATS group (MD -0.37, 95% CI -0.58 to -0.16). This finding suggests that mediastinal nodal station assessment may be a technical aspect requiring particular attention during NIVATS, although definitions and reporting methods varied across studies. Because mediastinal nodal station assessment can influence staging accuracy and subsequent adjuvant-treatment decisions, this finding should be regarded as a potential technical trade-off of NIVATS rather than a trivial difference.

R0 resection was reported in two studies and did not differ significantly between groups (RR 0.98, 95% CI 0.91-1.04). This result supports the interpretation that NIVATS did not clearly compromise macroscopic resection radicality in the included cohorts.

### Recurrence and other oncologic outcomes

Three studies reported recurrence or metastasis events in a format suitable for pooled analysis (**Figure 6**). NIVATS was associated with a borderline reduction in recurrence risk (RR 0.68, 95% CI 0.46-1.00; I² = 0.0%). Because recurrence definitions and follow-up durations differed across studies, this estimate should be interpreted cautiously. Individual studies also reported locoregional recurrence, distant recurrence, death during follow-up, chemotherapy delivery, and chemotherapy-related toxicity, but these outcomes were generally available from single studies and were therefore summarized narratively rather than pooled.

One study suggested that patients undergoing NIVATS may have better completion of planned adjuvant chemotherapy and lower severe treatment-related toxicity. Although this finding is clinically relevant, it was not sufficient for quantitative synthesis. It may nevertheless provide a potential link between perioperative recovery and longer-term oncologic management, particularly in patients requiring adjuvant treatment.

### Subgroup and meta-regression analyses

Exploratory subgroup analyses were performed by age, follow-up duration, region, sample size, and surgical scope. For OS, the pooled association appeared stronger in studies with mean age below 75 years, longer follow-up, mainland Chinese cohorts, and anatomical resections (**Supplementary Figure 1**). For DFS/RFS, similar patterns were observed, with more favorable estimates in longer follow-up and anatomical resection subgroups (**Supplementary Figure 2**). In contrast, estimates were less precise among elderly or mixed-resection cohorts.

Exploratory meta-regression did not identify statistically significant effect modification by age group, follow-up duration, sample size, surgical scope, or geographic region for either OS or DFS/RFS (**Supplementary Figure 3**). These analyses were limited by the small number of studies and should be interpreted as hypothesis-generating rather than confirmatory. Because lobar and sublobar resections differ in surgical indication, extent of lymph-node evaluation, and recurrence risk, surgical scope was considered a clinically relevant source of heterogeneity. However, studies with mixed resection types or without procedure-specific survival estimates limited definitive interpretation of the surgical-scope subgroup and meta-regression findings.

Prespecified subgroup analyses were conducted only when data permitted clinically meaningful and statistically estimable comparisons. Subgroup analyses were not performed for GGO/solid phenotype, pure or predominant GGO proportion, solid-component size, detailed tumor biology, pulmonary function, BMI, comorbidities, ASA/ECOG status, neoadjuvant/adjuvant therapy, or anesthetic subtype because these variables were incompletely reported, inconsistently defined, or available in fewer than two studies with usable survival HRs per subgroup. In particular, anesthetic protocols often overlapped across epidural/regional/local anesthesia with sedation, intravenous anesthesia, spontaneous ventilation, and non-intubated approaches, preventing reliable subtype classification. Propensity-score matching status was also not informative as a moderator because all studies contributing OS or DFS/RFS HRs used matching, weighting, or adjusted estimates. Subgroup analyses for secondary oncologic outcomes were not performed because of sparse studies per outcome and differing effect measures.

### Sensitivity and publication bias analyses

To test robustness, we repeated the primary pooling after excluding reconstructed or subgroup-derived survival estimates. The association remained stable for both OS (HR 0.64, 95% CI 0.47-0.89; P = 0.008) and DFS/RFS (HR 0.65, 95% CI 0.49-0.86; P = 0.002) (**Supplementary Figure 4**). Leave-one-out analysis confirmed that no single study materially altered the pooled effect for either endpoint (**Supplementary Figure 5**). Additional exploratory sensitivity analyses restricted to strict NSCLC-only primary survival data and analyses excluding high-selection special cohorts also showed broadly consistent associations; however, the latter analyses were interpreted cautiously because only two studies remained after exclusion (**Supplementary Figures 6**, **7**). Funnel plots and Egger’s test showed no evidence of significant small-study effects (OS, P = 0.757; DFS/RFS, P = 0.237), but these assessments were limited by the small number of studies (<10) and were therefore interpreted as exploratory (**Supplementary Figure 8**).

## Discussion

This systematic review and meta-analysis focused on the long-term prognosis of patients with NSCLC undergoing NIVATS compared with conventional intubated or mechanical ventilation VATS. The main finding is that NIVATS was associated with lower pooled hazards for OS and DFS/RFS, with a pooled HR of 0.66 for both endpoints. The results were stable in sensitivity analyses restricted to more robust survival estimates and were not accompanied by clear evidence of impaired total lymph node harvest or reduced R0 resection. However, these findings should not be interpreted as proof that non-intubation or spontaneous ventilation itself improves cancer survival. Given the predominance of retrospective studies and the likelihood that NIVATS was selectively offered to patients with more favorable clinical or tumor characteristics, the observed survival advantage is most plausibly explained, at least in part, by selection bias and residual confounding. The most defensible interpretation is that NIVATS appears not to compromise long-term oncologic outcomes in carefully selected NSCLC patients.

The long-term findings are clinically meaningful because the debate around NIVATS has often been centered on short-term safety. Many surgeons and anesthesiologists accept that NIVATS can reduce selected perioperative stressors, but remain cautious about its oncologic implications. Lung cancer surgery is fundamentally different from benign thoracic procedures because the quality of resection and lymph node staging can influence adjuvant treatment decisions, recurrence detection, and survival. The present analysis addresses this concern by evaluating survival outcomes together with nodal and margin-related endpoints. The absence of a significant reduction in total lymph node harvest and R0 resection provides an important context for interpreting the survival findings.

Several mechanisms may explain the observed association between NIVATS and long-term prognosis. Conventional intubated VATS requires airway instrumentation, neuromuscular blockade, and one-lung mechanical ventilation. These components may contribute to perioperative inflammation, oxidative stress, pulmonary injury, and transient immune dysfunction (44–46). In cancer surgery, the perioperative period is increasingly recognized as a biologically active interval during which surgical stress, inflammatory mediators, and suppression of cellular immunity may influence micrometastatic control (44, 45). By avoiding mechanical ventilation and reducing anesthetic invasiveness, NIVATS may attenuate this physiological disturbance. Some included studies reported lower postoperative inflammatory markers and better preservation of lymphocyte subsets after spontaneous ventilation VATS (31, 33–36), supporting this biological plausibility. However, these observations remain indirect; the current meta-analysis cannot prove that immune preservation is the causal pathway linking NIVATS to improved survival.

Another possible explanation is improved postoperative recovery. Faster recovery may facilitate earlier mobilization, better respiratory function, fewer complications, and greater tolerance of adjuvant therapy when indicated. The study addressing adjuvant chemotherapy suggested improved completion of planned treatment after non-intubated thoracic surgery (32). Although this evidence is currently limited to a single study, it raises an important point: the prognostic influence of NIVATS may not depend solely on intraoperative physiology, but also on the continuity of postoperative oncologic care. This hypothesis deserves further evaluation in cohorts with standardized reporting of adjuvant therapy indication, initiation, completion, and toxicity.

Compared with prior evidence syntheses, the present study adds a more focused oncologic perspective. Earlier reviews of non-intubated thoracoscopic surgery generally concluded that NIVATS was feasible and was associated with comparable or favorable perioperative outcomes, but they were limited by small numbers of lung-cancer studies, broad thoracic-disease populations, or endpoints centered on short-term recovery rather than OS or DFS/RFS (6–10). In contrast, our review restricted the clinical question to NSCLC or extractable lung-cancer oncologic data, used OS and DFS/RFS as primary endpoints, incorporated reconstructed or fixed-time survival estimates only with prespecified source classification and sensitivity analyses, and evaluated recurrence, R0 resection, and lymph-node assessment as supporting oncologic adequacy outcomes. Therefore, the main novelty is not simply showing that NIVATS can be performed safely, but synthesizing whether its potential perioperative advantages are accompanied by acceptable long-term cancer-related outcomes.

The finding regarding N2 station or group assessment deserves careful attention. Although total lymph node number, N1 node number, N2 node number, and R0 resection were not significantly different between groups, N2 station/group number was lower after NIVATS. This may reflect technical difficulty during mediastinal dissection under spontaneous ventilation, differences in surgical preference, or inconsistent definitions of nodal stations and groups across studies. It should not be overinterpreted as definitive evidence of inadequate mediastinal staging, especially because survival and recurrence outcomes did not suggest harm. Nevertheless, it identifies a practical boundary for NIVATS: oncologic standards must not be relaxed. Systematic or lobe-specific mediastinal nodal assessment should be maintained, and teams should have a low threshold for conversion when exposure, ventilation, bleeding, cough, or oncologic completeness becomes uncertain. In practical terms, even a small reduction in assessed mediastinal stations could theoretically increase the risk of understaging in individual patients and may influence the selection or omission of adjuvant therapy. This potential technical cost should be weighed against perioperative advantages when NIVATS is selected for cancer surgery.

Patient selection is central to interpreting these results. Most included studies were retrospective, and NIVATS was usually offered to selected patients at experienced centers. Such patients may have had smaller tumors, less advanced disease, better cardiopulmonary reserve, lower inflammatory burden, more favorable anatomy, a higher proportion of indolent radiologic phenotypes such as pure or predominant GGO lesions, or less aggressive tumor biology. Although many studies used propensity-score matching or multivariable adjustment, key unmeasured or inconsistently reported variables, including solid-component size, pure/predominant GGO proportion, subtle stage imbalance, molecular features, surgical selection, and institutional experience, could not be fully controlled. Therefore, the observed survival advantage should be interpreted as an association rather than proof of superiority, and selection bias remains the most likely explanation for at least part of the apparent benefit. Whether NIVATS itself produces any survival advantage requires prospective confirmation.

The subgroup analyses provide additional but limited insight. Survival associations appeared more evident in patients with mean age below 75 years, in studies with longer follow-up, and in anatomical resection cohorts. These patterns may indicate that the observed association becomes more apparent when follow-up is sufficient and when oncologic surgery is performed in a standardized manner. Conversely, estimates in elderly or mixed-resection populations were less precise, likely because of smaller sample size, competing mortality, heterogeneous surgical indications, and sparse events. The meta-regression analyses did not confirm statistically significant effect modification, which is unsurprising given the small number of studies. These subgroup findings should therefore be considered exploratory.

Our study has several strengths. First, it specifically focused on long-term prognosis rather than only perioperative outcomes, which better reflects the clinical priorities of NSCLC surgery. Second, OS and DFS/RFS were analyzed as primary endpoints, while recurrence, R0 resection, and lymph node metrics were evaluated as supporting oncologic adequacy outcomes. Third, sensitivity analyses were conducted to address the influence of reconstructed or subgroup-derived estimates. Fourth, the analysis distinguished lymph node number from lymph node station/group number, reducing the risk of combining clinically different indicators.

Several limitations should also be acknowledged. The most important limitation is the non-randomized nature of most included studies. Even after matching, residual confounding and selection bias remain possible. Second, survival outcomes were not reported uniformly. Some estimates were derived from figures or fixed-time survival rates and were therefore less reliable than directly reported HRs. Although sensitivity analyses supported the robustness of the main findings, this limitation should be considered when interpreting the pooled effect. Third, DFS and RFS were combined because terminology differed across studies; this approach increased statistical power but introduced endpoint heterogeneity. Fourth, several included cohorts represented special populations, such as elderly patients, patients with poor lung function, overweight patients, or patients after neoadjuvant therapy, which may limit generalizability. Fifth, the number of studies was small for recurrence, R0 resection, adjuvant treatment, and time-specific survival analyses. Sixth, definitions of lymph node stations, dissection protocols, conversion criteria, anesthetic management, and follow-up schedules were not standardized. Finally, radiologic tumor invasiveness markers, especially pure/predominant GGO proportion, solid tumor proportion, and solid-component size, were frequently not reported, and detailed tumor biological features were sparse. These missing variables are directly relevant to patient selection and prognosis, may partly or largely explain the observed survival association, and precluded reliable subgroup analysis or meta-regression based on radiologic phenotype or tumor biology.

The current evidence has practical implications. NIVATS should not be viewed as a simplified version of thoracic surgery, but as a specialized technique requiring close coordination between surgeons and anesthesiologists. It may be considered for selected NSCLC patients when the surgical team can maintain standard oncologic resection, ensure adequate nodal assessment, and convert promptly when needed. Future studies should move beyond feasibility and report standardized long-term outcomes. Ideally, prospective multicenter registries or randomized trials should define eligibility criteria, anesthetic protocols, nodal dissection requirements, conversion thresholds, adjuvant therapy delivery, and follow-up intervals in advance. Such designs are necessary to determine whether the survival association observed here represents a true biological or clinical benefit of NIVATS.

## Conclusions

In this systematic review and meta-analysis, NIVATS appeared not to compromise long-term oncologic outcomes compared with conventional intubated VATS in selected patients with NSCLC. Although pooled OS and DFS/RFS estimates favored NIVATS, this apparent survival advantage should be interpreted cautiously because the evidence is largely observational and vulnerable to selection bias and residual confounding. The finding of fewer N2 nodal stations or groups assessed after NIVATS also highlights the need to preserve mediastinal staging quality. NIVATS may be considered an oncologically feasible strategy when performed by experienced teams under strict selection criteria, but adequately powered prospective studies with standardized staging, surgical, anesthetic, and follow-up protocols are required before superiority can be inferred.

## Data Availability

The original contributions presented in the study are included in the article/**Supplementary Material**. Further inquiries can be directed to the corresponding authors.

## Supplementary material

Supplementary Figure 1 Subgroup analysis for overall survival. Exploratory OS subgroup analyses according to follow-up duration, mean age category, geographic region, sample size, and surgical scope. Subgroup analyses were performed only when each subgroup contained at least two studies.

Supplementary Figure 2 Subgroup analysis for DFS/RFS. Exploratory DFS/RFS subgroup analyses according to follow-up duration, mean age category, geographic region, sample size, and surgical scope. Subgroup analyses were performed only when each subgroup contained at least two studies.

Supplementary Figure 3 Exploratory meta-regression for primary survival outcomes. Meta-regression evaluated whether follow-up duration, mean age category, surgical scope, sample size, or geographic region modified the pooled OS or DFS/RFS estimates. These analyses were considered hypothesis-generating because of the small number of studies.

Supplementary Figure 4 Core-study-only sensitivity analysis for primary survival outcomes. Panel **(A)** shows the OS analysis and Panel **(B)** shows the DFS/RFS analysis after excluding reconstructed or subgroup-derived estimates.

Supplementary Figure 5 Leave-one-out sensitivity analysis for primary survival outcomes. Panel **(A)** shows pooled OS estimates and Panel **(B)** shows pooled DFS/RFS estimates after sequential omission of each included study, used to assess whether the pooled results were driven by any single study.

Supplementary Figure 6 Strict NSCLC-only sensitivity analysis for primary survival outcomes. Panel **(A)** shows OS and Panel **(B)** shows DFS/RFS restricted to strict NSCLC-only primary survival data using REML random-effects pooling.

Supplementary Figure 7 Sensitivity analysis after excluding high-selection special cohorts. Panel **(A)** shows OS and Panel **(B)** shows DFS/RFS after excluding the elderly mixed-resection cohort and the poor-lung-function cohort; these analyses were exploratory because only two studies remained.

Supplementary Figure 8 Funnel plots for primary survival outcomes. Panel **(A)** shows the funnel plot for OS and Panel **(B)** shows the funnel plot for DFS/RFS, used as exploratory assessments of small-study effects.

Supplementary Table 1 Study characteristics of the included studies with tumor/NSCLC-extractable counts, anesthetic technique and airway strategy, surgical procedure, tumor stage/profile, and extractable oncologic outcomes.

Supplementary Table 2 Baseline tumor characteristics, treatment-related factors, follow-up duration, and potential residual confounding in studies contributing to survival analyses.

Supplementary Table 3 Risk-of-bias assessment for the randomized study using RoB 2.

Supplementary Table 4 Newcastle-Ottawa Scale assessment for observational studies.

Supplementary Table 5 GRADE certainty of evidence. Outcome-level certainty assessment for primary and secondary outcomes.
