# Supplementary material for: Long-term oncologic outcomes after non-intubated versus intubated video-assisted thoracoscopic surgery for non-small cell lung cancer: a systematic review and meta-analysis
Source: Front Oncol. 2026 Aug 14;16:1933004. doi: 10.3389/fonc.2026.1933004 (PMC13521878; doi:10.3389/fonc.2026.1933004)
Supplement: Supplementary file 1 [file Supplementaryfile1.docx]

**Supplement File 1. Search strategies**

**Review topic:** Non-intubated or spontaneous-ventilation video-assisted thoracoscopic surgery versus intubated video-assisted thoracoscopic surgery for non-small cell lung cancer.

**Search date:** 30 June 2026.

**Note:** The main database searches were intentionally not restricted by outcome terms to avoid missing eligible comparative studies that did not report survival or nodal outcomes in the title or abstract. Outcome-focused terms were used for supplementary citation chasing and verification of secondary outcomes.

# PubMed/MEDLINE 141

| **Platform** | PubMed |
| --- | --- |
| **Date searched** | 30 June 2026 |
| **Fields** | Title/Abstract and MeSH terms |
| **Search strategy** | (("non-intubated"[Title/Abstract] OR nonintubated[Title/Abstract] OR "non intubated"[Title/Abstract] OR awake[Title/Abstract] OR tubeless[Title/Abstract] OR "spontaneous ventilation"[Title/Abstract] OR "spontaneously breathing"[Title/Abstract] OR "non-intubated thoracic surgery"[Title/Abstract] OR "nonintubated thoracic surgery"[Title/Abstract] OR NIVATS[Title/Abstract] OR NITS[Title/Abstract] OR "NI-VATS"[Title/Abstract] OR "SV-VATS"[Title/Abstract]) AND ("video-assisted thoracic surgery"[Title/Abstract] OR VATS[Title/Abstract] OR thoracoscop*[Title/Abstract] OR lobectom*[Title/Abstract] OR segmentectom*[Title/Abstract] OR "anatomic resection"[Title/Abstract] OR "anatomical resection"[Title/Abstract] OR "lung resection"[Title/Abstract] OR "pulmonary resection"[Title/Abstract]) AND ("non-small cell lung cancer"[Title/Abstract] OR "non-small-cell lung cancer"[Title/Abstract] OR NSCLC[Title/Abstract] OR "lung cancer"[Title/Abstract] OR "lung carcinoma"[Title/Abstract] OR "lung neoplasm"[Title/Abstract] OR "lung neoplasms"[MeSH Terms])) |

# Embase 184

| **Platform** | Embase.com alternative syntax |
| --- | --- |
| **Date searched** | 30 June 2026 |
| **Fields** | Title/abstract/keyword plus Emtree |
| **Search strategy** | (('non-intubated':ti,ab,kw OR nonintubated:ti,ab,kw OR 'non intubated':ti,ab,kw OR awake:ti,ab,kw OR tubeless:ti,ab,kw OR 'spontaneous ventilation':ti,ab,kw OR 'spontaneously breathing':ti,ab,kw OR 'non-intubated thoracic surgery':ti,ab,kw OR 'nonintubated thoracic surgery':ti,ab,kw OR nivats:ti,ab,kw OR nits:ti,ab,kw OR 'ni-vats':ti,ab,kw OR 'sv-vats':ti,ab,kw) AND ('video-assisted thoracic surgery':ti,ab,kw OR vats:ti,ab,kw OR thoracoscop*:ti,ab,kw OR lobectom*:ti,ab,kw OR segmentectom*:ti,ab,kw OR 'anatomic resection':ti,ab,kw OR 'anatomical resection':ti,ab,kw OR 'lung resection':ti,ab,kw OR 'pulmonary resection':ti,ab,kw) AND ('non-small cell lung cancer':ti,ab,kw OR 'non-small-cell lung cancer':ti,ab,kw OR nsclc:ti,ab,kw OR 'lung cancer':ti,ab,kw OR 'lung carcinoma':ti,ab,kw OR 'lung neoplasm':ti,ab,kw OR 'non small cell lung cancer'/exp OR 'lung cancer'/exp)) |

# Cochrane Library 43

| **Platform** | Cochrane CENTRAL |
| --- | --- |
| **Date searched** | 30 June 2026 |
| **Fields** | Title/Abstract/Keyword |
| **Search strategy** | #1 ("non-intubated" OR nonintubated OR "non intubated" OR awake OR tubeless OR "spontaneous ventilation" OR "spontaneously breathing" OR "non-intubated thoracic surgery" OR "nonintubated thoracic surgery" OR NIVATS OR NITS OR "NI-VATS" OR "SV-VATS"):ti,ab,kw #2 ("video-assisted thoracic surgery" OR VATS OR thoracoscop* OR lobectom* OR segmentectom* OR "anatomic resection" OR "anatomical resection" OR "lung resection" OR "pulmonary resection"):ti,ab,kw #3 ("non-small cell lung cancer" OR "non-small-cell lung cancer" OR NSCLC OR "lung cancer" OR "lung carcinoma" OR "lung neoplasm" OR "lung neoplasms"):ti,ab,kw #4 #1 AND #2 AND #3 |

# Web of Science Core Collection 181

| **Platform** | Web of Science Advanced Search |
| --- | --- |
| **Date searched** | 30 June 2026 |
| **Fields** | Topic |
| **Search strategy** | TS=(("non-intubated" OR nonintubated OR "non intubated" OR awake OR tubeless OR "spontaneous ventilation" OR "spontaneously breathing" OR "non-intubated thoracic surgery" OR "nonintubated thoracic surgery" OR NIVATS OR NITS OR "NI-VATS" OR "SV-VATS") AND ("video-assisted thoracic surgery" OR VATS OR thoracoscop* OR lobectom* OR segmentectom* OR "anatomic resection" OR "anatomical resection" OR "lung resection" OR "pulmonary resection") AND ("non-small cell lung cancer" OR "non-small-cell lung cancer" OR NSCLC OR "lung cancer" OR "lung carcinoma" OR "lung neoplasm*")) |

# ClinicalTrials.gov 639

| **Platform** | ClinicalTrials.gov |
| --- | --- |
| **Date searched** | 30 June 2026 |
| **Fields** | Other terms/condition |
| **Search strategy** | Condition/disease: lung cancer OR non-small cell lung cancer OR NSCLC Other terms: non-intubated OR nonintubated OR spontaneous ventilation OR tubeless OR awake OR NIVATS OR NITS OR VATS OR thoracoscopic OR lobectomy OR segmentectomy |

# Supplementary outcome-focused search

| **Platform** | Used for citation chasing and secondary-outcome verification |
| --- | --- |
| **Date searched** | 30 June 2026 |
| **Fields** | Adapted to each database |
| **Search strategy** | ("non-intubated" OR nonintubated OR "spontaneous ventilation" OR tubeless OR awake OR NIVATS OR NITS) AND (VATS OR thoracoscop* OR lobectom* OR segmentectom*) AND (NSCLC OR "non-small cell lung cancer" OR "lung cancer") AND ("overall survival" OR "disease-free survival" OR "recurrence-free survival" OR recurrence OR metastasis OR "lymph node" OR lymphadenectomy OR "lymph node station" OR "nodal station" OR "R0 resection" OR margin OR "adjuvant chemotherapy") |

**Recommended reporting statement**

A systematic search of PubMed/MEDLINE, Embase, the Cochrane Library, Web of Science Core Collection, and ClinicalTrials.gov was performed from database inception to 30 June 2026. The search combined terms for non-intubated or spontaneous-ventilation anesthesia, thoracoscopic lung resection, and lung cancer/NSCLC. No language or date restrictions were applied at the search stage. Reference lists of relevant reviews and included studies were also screened.
