# Supplementary material for: Long-term oncologic outcomes after non-intubated versus intubated video-assisted thoracoscopic surgery for non-small cell lung cancer: a systematic review and meta-analysis
Source: Front Oncol. 2026 Aug 14;16:1933004. doi: 10.3389/fonc.2026.1933004 (PMC13521878; doi:10.3389/fonc.2026.1933004)
Supplement: Supplementary file 2 [file Supplementaryfile2.docx]

| **Section and Topic** | **Item #** | **Checklist item** | **Location where item is reported** |
| --- | --- | --- | --- |
| **TITLE** | | |  |
| Title | 1 | Identify the report as a systematic review. | Manuscript p. 1. |
| **ABSTRACT** | | |  |
| Abstract | 2 | See the PRISMA 2020 for Abstracts checklist. | Manuscript pp. 2-4 (Abstract). |
| **INTRODUCTION** | | |  |
| Rationale | 3 | Describe the rationale for the review in the context of existing knowledge. | Manuscript pp. 4-6 (Introduction). |
| Objectives | 4 | Provide an explicit statement of the objective(s) or question(s) the review addresses. | Manuscript p. 6 (Introduction, objectives paragraph). |
| **METHODS** | | |  |
| Eligibility criteria | 5 | Specify the inclusion and exclusion criteria for the review and how studies were grouped for the syntheses. | Manuscript pp. 8-9 (Eligibility criteria; PICOS framework). |
| Information sources | 6 | Specify all databases, registers, websites, organisations, reference lists and other sources searched or consulted to identify studies. Specify the date when each source was last searched or consulted. | Manuscript p. 7 (Search strategy); Supplement File 1 cited in the supplementary file legends on p. 36. |
| Search strategy | 7 | Present the full search strategies for all databases, registers and websites, including any filters and limits used. | Supplement File 1; manuscript p. 7 and supplementary file legends p. 36. |
| Selection process | 8 | Specify the methods used to decide whether a study met the inclusion criteria of the review, including how many reviewers screened each record and each report retrieved, whether they worked independently, and if applicable, details of automation tools used in the process. | Manuscript p. 7 (screening by two reviewers) and pp. 15-16 (study selection results); Figure 1 legend p. 34. |
| Data collection process | 9 | Specify the methods used to collect data from reports, including how many reviewers collected data from each report, whether they worked independently, any processes for obtaining or confirming data from study investigators, and if applicable, details of automation tools used in the process. | Manuscript p. 10 (Data extraction). |
| Data items | 10a | List and define all outcomes for which data were sought. Specify whether all results that were compatible with each outcome domain in each study were sought (e.g. for all measures, time points, analyses), and if not, the methods used to decide which results to collect. | Manuscript pp. 9-10 (Outcomes) and pp. 11-14 (data handling/statistical analysis). |
|  | 10b | List and define all other variables for which data were sought (e.g. participant and intervention characteristics, funding sources). Describe any assumptions made about any missing or unclear information. | Manuscript p. 10 (Data extraction); Supplementary Table S1 legend p. 37. |
| Study risk of bias assessment | 11 | Specify the methods used to assess risk of bias in the included studies, including details of the tool(s) used, how many reviewers assessed each study and whether they worked independently, and if applicable, details of automation tools used in the process. | Manuscript p. 11 (Risk of bias assessment) and pp. 18-19 (risk-of-bias results); Supplementary Tables S3-S4 legends p. 37. |
| Effect measures | 12 | Specify for each outcome the effect measure(s) (e.g. risk ratio, mean difference) used in the synthesis or presentation of results. | Manuscript pp. 11-14 (Statistical analysis). |
| Synthesis methods | 13a | Describe the processes used to decide which studies were eligible for each synthesis (e.g. tabulating the study intervention characteristics and comparing against the planned groups for each synthesis (item #5)). | Manuscript pp. 8-9 (Eligibility criteria) and pp. 15-18 (study grouping/characteristics). |
|  | 13b | Describe any methods required to prepare the data for presentation or synthesis, such as handling of missing summary statistics, or data conversions. | Manuscript pp. 11-14 (data transformation, KM reconstruction, and fixed-time survival methods). |
|  | 13c | Describe any methods used to tabulate or visually display results of individual studies and syntheses. | Manuscript pp. 19-25 (Results); Figure 2-Figure 6 legends pp. 34-35; Supplementary Figure 1-Supplementary Figure 8 legends pp. 35-36. |
|  | 13d | Describe any methods used to synthesize results and provide a rationale for the choice(s). If meta-analysis was performed, describe the model(s), method(s) to identify the presence and extent of statistical heterogeneity, and software package(s) used. | Manuscript pp. 14-15 (REML random-effects model, heterogeneity assessment, and software). |
|  | 13e | Describe any methods used to explore possible causes of heterogeneity among study results (e.g. subgroup analysis, meta-regression). | Manuscript p. 14 and pp. 22-24; Supplementary Figures 1-3 legends pp. 35-36. |
|  | 13f | Describe any sensitivity analyses conducted to assess robustness of the synthesized results. | Manuscript p. 14 and pp. 24-25; Supplementary Figures 4-7 legends p. 36. |
| Reporting bias assessment | 14 | Describe any methods used to assess risk of bias due to missing results in a synthesis (arising from reporting biases). | Manuscript pp. 14-15 and pp. 24-25; Supplementary Figure 8 legend p. 36. |
| Certainty assessment | 15 | Describe any methods used to assess certainty (or confidence) in the body of evidence for an outcome. | Manuscript p. 11 (GRADE methods) and pp. 18-19 (GRADE results); Supplementary Table S5 legend p. 37. |
| **RESULTS** | | |  |
| Study selection | 16a | Describe the results of the search and selection process, from the number of records identified in the search to the number of studies included in the review, ideally using a flow diagram. | Manuscript pp. 15-16; Figure 1 legend p. 34. |
|  | 16b | Cite studies that might appear to meet the inclusion criteria, but which were excluded, and explain why they were excluded. | Manuscript pp. 15-16; Figure 1 legend p. 34. |
| Study characteristics | 17 | Cite each included study and present its characteristics. | Manuscript pp. 15-18; Supplementary Table S1 legend p. 37; included-study references [27-43] pp. 40-43. |
| Risk of bias in studies | 18 | Present assessments of risk of bias for each included study. | Manuscript pp. 18-19; Supplementary Tables S3-S4 legends p. 37. |
| Results of individual studies | 19 | For all outcomes, present, for each study: (a) summary statistics for each group (where appropriate) and (b) an effect estimate and its precision (e.g. confidence/credible interval), ideally using structured tables or plots. | Manuscript pp. 19-25; Figures 2-6 legends pp. 34-35; Supplementary Figures 1-8 legends pp. 35-36. |
| Results of syntheses | 20a | For each synthesis, briefly summarise the characteristics and risk of bias among contributing studies. | Manuscript pp. 15-25; risk-of-bias/certainty summary pp. 18-19; Supplementary Tables S3-S5 legends p. 37. |
|  | 20b | Present results of all statistical syntheses conducted. If meta-analysis was done, present for each the summary estimate and its precision (e.g. confidence/credible interval) and measures of statistical heterogeneity. If comparing groups, describe the direction of the effect. | Manuscript pp. 19-25; Figures 2-6 legends pp. 34-35; Supplementary Figures 1-8 legends pp. 35-36. |
|  | 20c | Present results of all investigations of possible causes of heterogeneity among study results. | Manuscript pp. 22-24; Supplementary Figures 1-3 legends pp. 35-36. |
|  | 20d | Present results of all sensitivity analyses conducted to assess the robustness of the synthesized results. | Manuscript pp. 24-25; Supplementary Figures 4-7 legends p. 36. |
| Reporting biases | 21 | Present assessments of risk of bias due to missing results (arising from reporting biases) for each synthesis assessed. | Manuscript pp. 24-25; Supplementary Figure 8 legend p. 36. |
| Certainty of evidence | 22 | Present assessments of certainty (or confidence) in the body of evidence for each outcome assessed. | Manuscript p. 3 (Abstract results) and pp. 18-19 (certainty results); Supplementary Table S5 legend p. 37. |
| **DISCUSSION** | | |  |
| Discussion | 23a | Provide a general interpretation of the results in the context of other evidence. | Manuscript pp. 25-27 (Discussion: interpretation and comparison with prior evidence). |
|  | 23b | Discuss any limitations of the evidence included in the review. | Manuscript pp. 28-31 (Discussion: limitations of included evidence). |
|  | 23c | Discuss any limitations of the review processes used. | Manuscript pp. 30-31 (Discussion: limitations of review processes and evidence synthesis). |
|  | 23d | Discuss implications of the results for practice, policy, and future research. | Manuscript pp. 31-33 (Discussion: implications; Conclusions). |
| **OTHER INFORMATION** | | |  |
| Registration and protocol | 24a | Provide registration information for the review, including register name and registration number, or state that the review was not registered. | Manuscript p. 7; PROSPERO CRD420261437783. |
|  | 24b | Indicate where the review protocol can be accessed, or state that a protocol was not prepared. | Manuscript p. 7; PROSPERO CRD420261437783. |
|  | 24c | Describe and explain any amendments to information provided at registration or in the protocol. | Not applicable; no protocol amendments were reported in the manuscript. |
| Support | 25 | Describe sources of financial or non-financial support for the review, and the role of the funders or sponsors in the review. | Manuscript p. 33 (Funding). |
| Competing interests | 26 | Declare any competing interests of review authors. | Manuscript p. 33 (Competing interests). |
| Availability of data, code and other materials | 27 | Report which of the following are publicly available and where they can be found: template data collection forms; data extracted from included studies; data used for all analyses; analytic code; any other materials used in the review. | Manuscript p. 33 (Availability of data and materials); Supplement File 2 and Supplement File 3 legends p. 37. |

*From:*  Page MJ, McKenzie JE, Bossuyt PM, Boutron I, Hoffmann TC, Mulrow CD, et al. The PRISMA 2020 statement: an updated guideline for reporting systematic reviews. BMJ 2021;372:n71. doi: 10.1136/bmj.n71. This work is licensed under CC BY 4.0. To view a copy of this license, visit <https://creativecommons.org/licenses/by/4.0/>
