# Supplementary material for: Long-term oncologic outcomes after non-intubated versus intubated video-assisted thoracoscopic surgery for non-small cell lung cancer: a systematic review and meta-analysis
Source: Front Oncol. 2026 Aug 14;16:1933004. doi: 10.3389/fonc.2026.1933004 (PMC13521878; doi:10.3389/fonc.2026.1933004)
Supplement: Supplementary file 3 [file Table1.docx]

Supplementary Table S1. Study characteristics of the 17 included studies with tumor-only counts

| **Study** | **Country/region** | **Study design** | **Population/eligibility** | **Experimental group** | **Control group** | **Anesthetic technique and airway strategy** | **Experimental, n** | **Control, n** | **Total tumor n** | **Age** | **ASA/ECOG status** | **Surgical procedure** | **Tumor stage/profile** | **Extracted oncologic outcomes** |
| --- | --- | --- | --- | --- | --- | --- | --- | --- | --- | --- | --- | --- | --- | --- |
| Chen et al., 2011 | China | Retrospective cohort | Clinical stage I-II peripheral NSCLC undergoing thoracoscopic lobectomy | Non-intubated VATS under thoracic epidural/local anesthesia with spontaneous ventilation | Intubated VATS under general anesthesia with one-lung ventilation | NIVATS: thoracic epidural anesthesia plus propofol/fentanyl sedation with spontaneous ventilation; oxygen delivered through a ventilation mask; no laryngeal mask airway (LMA) reported. IVATS: general anesthesia with double-lumen endotracheal intubation and one-lung ventilation. | 30 | 30 | 60 | 57.9 +/- 10.4 / 56.5 +/- 9.5 | ASA I 1/2, II 21/16, III 8/12 | VATS lobectomy | Predominantly pathological stage IA | Total lymph nodes |
| Wu et al., 2013 | China | Retrospective cohort | Geriatric patients aged >=65 years with clinical stage I-II NSCLC | Non-intubated VATS with spontaneous ventilation | Intubated VATS with general anesthesia | NIVATS: thoracic epidural anesthesia, intrathoracic vagal blockade, and propofol/fentanyl sedation with spontaneous ventilation; oxygen delivered through a ventilation mask; no LMA reported. IVATS: double-lumen endotracheal intubation with single-lung ventilation. | 36 | 48 | 84 | 72.9 (65-84) / 73.0 (65-87) | ASA II 11/11, III 23/37, IV 2/0 | VATS lobectomy | Pathological stage I-IIIA | Total lymph nodes |
| Liu et al., 2016 | China | Retrospective PSM cohort | NSCLC patients undergoing VATS anatomical resection | Non-intubated intravenous anesthesia with spontaneous ventilation VATS | Intubated anesthesia with single-lung ventilation VATS | NIVATS: non-intubated intravenous anesthesia with spontaneous ventilation and intrathoracic vagal blockade; specific intraoperative airway device not reported; mask-assisted ventilation was described after wound closure. IVATS: intubated anesthesia with single-lung mechanical ventilation. | 136 | 136 | 272 | Matched subgroup ages reported | Eligibility ASA <=3 | VATS segmentectomy or lobectomy | Segmentectomy stage I; lobectomy stage I-III | Lymph node number and station number |
| Guo et al., 2016 | China | Retrospective cohort | Thoracoscopic segmentectomy cohort; malignant and benign lesions included | Non-intubated thoracoscopic segmentectomy | Intubated thoracoscopic segmentectomy | NIVATS: thoracic epidural anesthesia, intrathoracic vagal blockade, and sedation with spontaneous ventilation; LMA used for supplemental oxygen. IVATS: intubated general anesthesia with double-lumen tube ventilation. | 39 | 61 | 100 | 49.10 +/- 12.78 / 56.63 +/- 12.70 | ASA I 12/14, II 33/65, III 3/13 | VATS segmentectomy | Mixed disease; malignant 39/48 vs 61/92 | Total lymph nodes reported for primary NSCLC patients, but NSCLC-only denominator/baseline not extractable |
| AlGhamdi et al., 2018 | South Korea | Retrospective cohort | Lung cancer/NSCLC patients undergoing VATS lobectomy | Non-intubated VATS lobectomy with spontaneous ventilation | Intubated VATS lobectomy with general anesthesia | NIVATS: propofol/dexmedetomidine sedation with spontaneous ventilation plus thoracoscopic intercostal and vagal nerve block; capnometry via nostril; LMA not reported. IVATS: intubated general anesthesia with one-lung ventilation. | 30 | 30 | 60 | 64.9 +/- 10.5 / 66.1 +/- 9.5 | NR | VATS lobectomy | Pathological IA-IIIA | Total lymph nodes |
| Furak et al., 2020 | Hungary | Retrospective cohort | Stage IB-IIIB NSCLC receiving adjuvant chemotherapy after VATS lobectomy | Non-intubated VATS lobectomy followed by planned adjuvant chemotherapy | Intubated VATS lobectomy followed by planned adjuvant chemotherapy | NIVATS/NITS: target-controlled propofol anesthesia with BIS 40-60; LMA inserted for airway maintenance; spontaneous breathing maintained. IVATS: general anesthesia with double-lumen endotracheal intubation. | 28 | 38 | 66 | 63.03 / 64 years | ECOG 0 19/28 vs 28/38; ECOG 1 9/28 vs 10/38 | VATS lobectomy | Stage IB-IIIB NSCLC | Adjuvant chemotherapy completion/toxicity; survival status |
| Wang et al., 2021 | China | Retrospective PSM cohort | Clinical stage I NSCLC undergoing thoracoscopic lobectomy | Non-intubated VATS lobectomy | Intubated VATS lobectomy | NIVATS: spontaneous ventilation under fentanyl/propofol target-controlled sedation; regional anesthesia by thoracic epidural block or intrathoracic intercostal block plus vagal blockade; oxygen via ventilation mask; no LMA reported. IVATS: intubated general anesthesia with one-lung ventilation. | 97 | 97 | 194 | Matched cohort | NR | VATS lobectomy | Clinical stage I NSCLC | OS HR; recurrence/FFR HR; recurrence pattern |
| Zheng et al., 2021 | China | Retrospective PSM cohort | Invasive NSCLC undergoing VATS lobectomy and lymph node dissection | Spontaneous-ventilation VATS lobectomy | Mechanical-ventilation VATS lobectomy | SV-VATS: spontaneous ventilation under intravenous propofol sedation without muscle relaxant; double-lumen LMA commonly used, with nasal cannula or facial mask used in selected situations. MV-VATS: double-lumen endotracheal intubation with mechanical ventilation. | 200 | 200 | 400 | Matched cohort | NR | VATS lobectomy | Pathological stage I-III invasive NSCLC | OS HR; DFS HR; N1/N2 node and station data |
| Chen et al., 2022 | China | Retrospective PSM cohort | Patients aged >=75 years with pathologically confirmed NSCLC | Non-intubated thoracoscopic surgery | Intubated thoracoscopic surgery | NIVATS: thoracic epidural anesthesia or intercostal nerve block plus intrathoracic vagal block with target-controlled sedation; oxygen support by ventilation mask or high-flow nasal cannula; no tracheal intubation. IVATS: general anesthesia with double-lumen endotracheal intubation. | 79 | 158 | 237 | 79.4 +/- 3.7 / 79.3 +/- 3.6 | NR | Mixed thoracoscopic resection | Predominantly stage I-III elderly NSCLC | OS HR; RFS HR; recurrence/death events; lymph nodes; margin |
| Wang et al., 2022b | China | Retrospective PSM cohort | NSCLC patients with poor lung function undergoing VATS anatomical resection | Spontaneous-ventilation VATS anatomical resection | Mechanical-ventilation VATS anatomical resection | SV-VATS: intravenous anesthesia with spontaneous ventilation using a third-generation double-tube LMA, thoracic paravertebral block, visceral pleural surface anesthesia, and thoracic vagus nerve block; manual ventilation/SIMV allowed if needed. MV-VATS: double-lumen intubated mechanical ventilation. | 31 | 62 | 93 | Categorical age reported | ASA I 0/1, II 31/57, III 0/4 | VATS anatomical resection | Mixed TNM stages | OS/DFS HR; N1/N2 node and station data |
| Wang et al., 2022a | China | Retrospective PSM cohort | Patients aged >=65 years with NSCLC undergoing SV-VATS or MV-VATS | Spontaneous-ventilation VATS | Mechanical-ventilation VATS | SV-VATS: intravenous anesthesia with LMA-supported spontaneous ventilation, thoracic paravertebral block, visceral pleural surface anesthesia, and thoracic vagus nerve block. MV-VATS: general endotracheal anesthesia with mechanical ventilation. | 80 | 80 | 160 | 70.0 +/- 4.5 / 70.1 +/- 4.4 | ASA I 0/1, II 71/70, III 9/9 | Lobectomy, segmentectomy, or wedge resection | Mixed T/N/M; after PSM mostly M0 | Total, N1, and N2 lymph node numbers |
| Wu et al., 2020 | China | Retrospective PSM cohort | NSCLC patients with BMI >=25 kg/m2 undergoing primary lung cancer resection | Spontaneous-ventilation VATS | Mechanical-ventilation/intubated VATS | SV-VATS: intravenous anesthesia with LMA-supported spontaneous ventilation, thoracic paravertebral block, visceral pleural surface anesthesia, and thoracic vagus nerve block; epidural block if necessary. MV-VATS: double-lumen intubated mechanical ventilation. | 48 | 48 | 96 | Categorical age reported | ASA I-II 48/48, III 0/0 | Mixed VATS resection | Mixed TNM; after PSM all M0 | Total, N1, and N2 lymph node numbers |
| Guo et al., 2023 | China | Randomized clinical study | Early-stage NSCLC patients undergoing thoracoscopic surgery | Spontaneous-breathing uniportal VATS | Conventional intubated thoracoscopic surgery | NIVATS: spontaneous-breathing uniportal VATS under sedative/analgesic anesthesia with paravertebral, pleural surface, and vagal nerve block; specific airway device not reported. IVATS: conventional double-lumen tracheal intubation with single-lung mechanical ventilation. | 41 | 41 | 82 | 58.68 +/- 5.99 / 58.00 +/- 8.05 | NR | Thoracoscopic lung cancer surgery | Early-stage NSCLC | 1-year OS, chemotherapy rate, recurrence/metastasis |
| Yu et al., 2024 | Thailand | Retrospective cohort/IPTW | Lung cancer patients undergoing lobectomy with lymph node dissection | Non-intubated VATS lobectomy | Intubated VATS lobectomy | NIVATS: fentanyl/propofol titration without inhalational anesthetic or muscle relaxant; spontaneous ventilation maintained with oxygen via facemask and reservoir bag; intercostal and vagal nerve block; no LMA reported. IVATS/GAVATS: general anesthesia with double-lumen intubation. | 50 | 78 | 128 | 65 (55.5-72) / 65 (59-73) | ASA >3 excluded | VATS lobectomy | Lung cancer, not pure NSCLC; rare small cell/carcinoid/carcinosarcoma present | Median lymph node harvest/stations reported only for whole lung-cancer cohort |
| Zhao et al., 2024 | China | Retrospective PSM non-inferiority cohort | NSCLC patients aged >80 years undergoing VATS | Spontaneous-ventilation/non-intubated VATS | Mechanical-ventilation/intubated VATS | SV-VATS: propofol/remifentanil/dexmedetomidine anesthesia with LMA-supported spontaneous ventilation; intercostal nerve block and thoracic vagus nerve block were used. MV-VATS: double-lumen endotracheal intubation with mechanical ventilation. | 22 | 44 | 66 | 82.5 +/- 1.95 / 82.5 +/- 2.12 | Matched | Mixed VATS resection | Octogenarian NSCLC | OS/DFS KM curves |
| Udelsman et al., 2024 | United States | Retrospective PSM cohort | Mixed thoracoscopic surgery cohort; primary lung cancer subgroup available | Non-intubated thoracoscopic surgery | Intubated thoracoscopic surgery | NIVATS: monitored sedation with propofol, midazolam, and fentanyl; spontaneous ventilation maintained; oxygen delivered through a face mask or LMA, with preemptive LMA in 7/72 (9.7%); no bronchial blocker. IVATS: standard double-lumen endotracheal intubation. | 30 | 60 | 90 | Overall matched cohort about 67.9/68.2 | Matched on ASA/comorbidities | Mixed thoracoscopic procedures | Mixed indications; not NSCLC-only | 3-year OS |
| Zheng et al., 2026 | China | Retrospective PSM cohort | NSCLC patients undergoing surgery after neoadjuvant therapy | Non-intubated VATS after neoadjuvant therapy | Intubated VATS after neoadjuvant therapy | NI-VATS: LMA-supported spontaneous ventilation with BIS-guided sedation, thoracic paravertebral block, and operative-side vagal nerve block. I-VATS: double-lumen endotracheal intubation with general anesthesia. | 31 | 31 | 62 | 63.71 +/- 7.21 / 63.68 +/- 8.64 | NR | VATS lobectomy after neoadjuvant therapy | Stage II-III NSCLC | Total nodes, station/groups, R0 resection |

**Note:** Counts refer to tumor/NSCLC-extractable patients included in qualitative synthesis or outcome extraction. For studies with mixed benign/malignant disease, mixed thoracic indications, or rare non-NSCLC histologies, benign cases, non-oncologic thoracic indications, and non-target histologies were not counted when extractable target-population data were available. Experimental group refers to non-intubated/spontaneous-ventilation VATS; control group refers to conventional intubated/general-anesthesia VATS with mechanical or one-lung ventilation. Age, ASA/ECOG status, tumor stage, and surgical procedures are presented as reported in the original studies. Extracted oncologic outcomes indicate data available for this review and do not imply that every outcome was included in quantitative pooling. Abbreviations: ASA, American Society of Anesthesiologists physical status; DFS, disease-free survival; ECOG, Eastern Cooperative Oncology Group; GAVATS, general-anesthesia video-assisted thoracoscopic surgery; IVATS, intubated video-assisted thoracoscopic surgery; NIVATS, non-intubated video-assisted thoracoscopic surgery; NSCLC, non-small cell lung cancer; OS, overall survival; PSM, propensity-score matching; RFS, recurrence-free survival; VATS, video-assisted thoracoscopic surgery.
