# Supplementary material for: Long-term oncologic outcomes after non-intubated versus intubated video-assisted thoracoscopic surgery for non-small cell lung cancer: a systematic review and meta-analysis
Source: Front Oncol. 2026 Aug 14;16:1933004. doi: 10.3389/fonc.2026.1933004 (PMC13521878; doi:10.3389/fonc.2026.1933004)
Supplement: Supplementary file 4 [file Table2.docx]

Supplementary Table S2. Baseline tumor characteristics, treatment-related factors, follow-up duration, and potential residual confounding in studies contributing to survival analyses

| **Study** | **Survival outcome contribution** | **Matched/analysis cohort** | **ASA/ECOG or comorbidity status** | **Surgical procedure/scope** | **Clinical/study stage distribution** | **Pathological or reported TNM distribution** | **Radiologic GGO/solid phenotype** | **Pure/predominant GGO proportion** | **Tumor size/solid component** | **Tumor biology/pathologic factors** | **Follow-up duration** | **Matching/adjustment variables relevant to comparability** | **Residual confounding/comparability comment** |
| --- | --- | --- | --- | --- | --- | --- | --- | --- | --- | --- | --- | --- | --- |
| Wang et al., 2021 | OS and freedom from recurrence/DFS-RFS | NIVATS lobectomy n=97; intubated lobectomy n=97 after PSM | NR | VATS lobectomy | All patients had clinical stage I unilateral lung cancer (clinical IA/IB eligible); detailed clinical IA vs IB distribution after matching was not reported. | Pathological stage after PSM: NIVATS IA1 13 (13%), IA2 45 (47%), IA3 29 (30%), IB 10 (10%); intubated IA1 15 (16%), IA2 37 (38%), IA3 31 (32%), IB 14 (14%). | NR for pure/predominant GGO or solid-tumor proportion. | Not reported | Tumor size: NIVATS 19.8 +/- 7.8 mm vs IVATS 20.0 +/- 8.4 mm; solid-component size NR. | Adenocarcinoma 95/97 (97%) vs 89/97 (92%); squamous 0 vs 4/97; carcinoid 1/97 vs 1/97; rare histologies reported. | Mean follow-up: NIVATS 74.5 months (SD 1.6); intubated 73.9 months (SD 2.2). Maximum follow-up 107 months. | Age, sex, BMI, smoking history, preoperative comorbidities, previous cancer history, pulmonary function, and cancer stage (IA vs IB). | Stage and tumor size were well balanced after PSM. However, radiologic invasiveness, GGO/solid phenotype, solid-component size, and molecular features were not reported; rare non-NSCLC/low-grade histologies were present and survival estimates were not histology-stratified. |
| Zheng et al., 2021 | OS and DFS | SV-VATS n=200; MV-VATS n=200 after 1:1 PSM | NR | VATS lobectomy | Invasive NSCLC stage I-III; stage IV and non-invasive lesions were excluded. Clinical stage distribution was not separately reported. | Reported TNM after PSM: SV-VATS stage I 150 (75.0%), II 19 (9.5%), III 31 (15.5%); MV-VATS stage I 160 (80.0%), II 13 (6.5%), III 27 (13.5%). T stage: SV T1 124, T2 68, T3/T4 8; MV T1 128, T2 62, T3/T4 10. N stage: SV N0 154, N1 17, N2 29; MV N0 167, N1 9, N2 24. | AIS excluded; pure/predominant GGO and solid-tumor proportions NR. | Not reported | Group-level tumor size and solid-component size NR. | Pathology after PSM: adenocarcinoma 176/200 vs 188/200, squamous 15/200 vs 6/200, others 9/200 vs 6/200. | Median follow-up 4.78 years (IQR, 3.78-6.62 years); follow-up range not reported. | Age, BMI, sex, TNM stage, smoking status, and personal history in the propensity model. | Reported TNM variables were included in PSM and were broadly comparable, but CT density, GGO/solid phenotype, solid-component size, and molecular features were unavailable; residual confounding related to tumor biology and institutional selection remains possible. |
| Chen et al., 2022 | OS and RFS | Non-intubated VATS n=79; intubated VATS n=158 after PSM | NR | Mixed thoracoscopic resection | Disease stage after PSM: intubated I 137 (86.7%), II 13 (8.2%), III 8 (5.1%); non-intubated I 69 (87.3%), II 6 (7.6%), III 4 (5.1%). | Pathological T stage after PSM: intubated pT1 98 (62.0%), pT2 49 (31.0%), pT3 11 (7.0%); non-intubated pT1 50 (63.3%), pT2 24 (30.4%), pT3 5 (6.3%). Pathological N stage: intubated pN0 149 (94.3%), pN1 5 (3.2%), pN2 4 (2.5%); non-intubated pN0 76 (96.2%), pN1 1 (1.3%), pN2 2 (2.5%). | NR for pure/predominant GGO or solid-tumor proportion. | Not reported | NR for tumor size and solid-component size. | Differentiation well 16/79 vs 29/158; VPI 20/79 vs 50/158; LVI 19/79 vs 26/158; adenocarcinoma 66/79 vs 117/158; SqCC 9/79 vs 18/158; adenosquamous 3/79 vs 17/158. | Median follow-up for all matched patients 45.5 months; follow-up range not reported. | Age, sex, BMI, family history, Charlson comorbidity index, pulmonary function (FEV1/FVC), and disease stage. | Both groups were predominantly stage I after matching, but surgical extent differed, with more wedge resections in the NIVATS group and more lobectomies in the intubated group. Radiologic GGO/solid phenotype and molecular features were not reported; residual confounding by frailty, tumor phenotype, and surgical extent remains possible. |
| Wang et al., 2022b | OS and DFS | SV-VATS n=31; MV-VATS n=62 after 1:2 PSM in patients with poor lung function | ASA I 0/1, II 31/57, III 0/4 | VATS anatomical resection | Preoperative T/N/M stage was reported as a confounder. Clinical TNM stage grouping was not provided. | After PSM, reported T stage: SV T1 2 (6%), T2 17 (55%), T3 11 (36%), T4 1 (3%); MV T1 39 (63%), T2 20 (32%), T3 1 (2%), T4 2 (3%). Reported N stage: SV N0 22 (71%), N1 1 (3%), N2 7 (23%), N3 1 (3%); MV N0 43 (69%), N1 5 (8%), N2 13 (21%), N3 1 (2%). Reported M stage: SV M0 28 (90%), M1 3 (10%); MV M0 58 (91%), M1 4 (9%). Pathological stage grouping was not separately reported. | NR for pure/predominant GGO or solid-tumor proportion. | Not reported | Eligibility tumor size <=5 cm; group-level tumor size and solid-component size NR. | PSM included histological analysis, but group-level histology distribution not fully extractable from main text; NR in table. | Median follow-up: SV-VATS 46.83 months; MV-VATS 50.97 months; follow-up range not reported. | Sex, age, tumor location, T stage, N stage, M stage, ASA status class, and surgical technique. | This poor-lung-function cohort was matched for reported clinical variables, but the reported post-PSM T-stage distribution showed marked imbalance or possible reporting inconsistency. Radiologic tumor phenotype and solid-component size were unavailable; pulmonary reserve and stage imbalance may confound survival estimates. |
| Zhao et al., 2024 | OS and DFS | SV-VATS n=22; MV-VATS n=44 after 1:2 PSM in octogenarian NSCLC patients | Matched | Mixed VATS resection | The main article described early-stage NSCLC and included tumors <=5 cm; clinical stage distribution was not reported in the main text. | Online Supplementary Table S1 after PSM: MV-VATS T1 33 (75.0%), T2/T3 11 (25.0%); SV-VATS T1 15 (68.2%), T2/T3 7 (31.8%). N stage: MV N0 41 (93.2%), N1/N2 3 (6.8%); SV N0 21 (95.5%), N1/N2 1 (4.5%). TNM stage: MV stage I 37 (84.1%), stage II/III 7 (15.9%); SV stage I 19 (86.4%), stage II/III 3 (13.6%). | NR for pure/predominant GGO or solid-tumor proportion. | Not reported | Eligibility tumor size <=5 cm; group-level tumor size and solid-component size NR. | Detailed pathological characteristics were not reported in the available full text or supplementary material. | Median follow-up 23.8 months; follow-up range not reported. | Age, sex, BMI, ASA status class, tumor position, and surgical type. | This small octogenarian cohort was predominantly stage I after PSM, with short follow-up. Radiologic GGO/solid phenotype, solid-component size, and detailed pathological features were not reported; competing mortality and mixed surgical procedures may contribute to residual confounding. |
| Udelsman et al., 2024 | OS only (3-year survival; primary lung cancer subgroup) | Non-intubated thoracoscopic surgery n=30; intubated thoracoscopic surgery n=60 after separate 2:1 PSM for primary lung cancer | Matched on ASA/comorbidities | Mixed thoracoscopic procedures | Clinical T status: intubated cT1 51 (85%), cT2 8 (13%), cT3 1 (2%); non-intubated cT1 27 (90%), cT2 2 (7%), cT3 1 (3%). Clinical N status: intubated cN0 59 (98%), cN1 1 (2%); non-intubated cN0 30 (100%), cN1 0. | Pathologic T status: intubated pT1 44 (73%), pT2 12 (20%), pT3 4 (7%), pT4 0; non-intubated pT1 24 (80%), pT2 4 (13%), pT3 1 (3%), pT4 1 (3%). Pathologic N status: intubated pN0 52 (87%), pN1 5 (8%), pN2 2 (3%), pNX 1 (2%); non-intubated pN0 25 (83%), pN1 0, pN2 1 (3%), pNX 4 (13%). | NR for pure/predominant GGO or solid-tumor proportion. | Not reported | NR for tumor size and solid-component size. | Primary lung cancer indication reported; NSCLC-specific histology/molecular features NR in extractable subgroup. | Median follow-up in the overall matched cohort: intubated 30.6 months; non-intubated 36.0 months. Primary lung cancer subgroup survival was reported at 3 years; range not reported. | Age, sex, BMI, ASA score, comorbidities, smoking status, predicted FEV1, prior cardiothoracic surgery, procedure type, and oncologic indication; separate PSM for primary lung cancer subgroup. | Clinical and pathological stage were explicitly reported and mostly early-stage. Although the analysis used the primary lung cancer subgroup, NSCLC-specific histology was not fully separable, pNX was more frequent in the non-intubated group, and radiologic GGO/solid features were not reported. |
