# Supplementary material for: Long-term oncologic outcomes after non-intubated versus intubated video-assisted thoracoscopic surgery for non-small cell lung cancer: a systematic review and meta-analysis
Source: Front Oncol. 2026 Aug 14;16:1933004. doi: 10.3389/fonc.2026.1933004 (PMC13521878; doi:10.3389/fonc.2026.1933004)
Supplement: Supplementary file 5 [file Table3.docx]

Supplementary Table S3. Risk-of-bias assessment for the randomized study using RoB 2

| **Study** | **Design** | **D1** | **D2** | **D3** | **D4** | **D5** | **Overall risk of_bias** |
| --- | --- | --- | --- | --- | --- | --- | --- |
| Guo 2023 | Randomized clinical study | Some concerns | Some concerns | Low risk | Some concerns | Some concerns | Some concerns |

**Note:** Risk of bias for the randomized study was assessed using the Cochrane RoB 2 tool. D1 indicates bias arising from the randomization process; D2, bias due to deviations from intended interventions; D3, bias due to missing outcome data; D4, bias in measurement of the outcome; and D5, bias in selection of the reported result. The overall judgment follows the RoB 2 domain-level algorithm. Judgments are reported as low risk, some concerns, or high risk.
