# Supplementary material for: Long-term oncologic outcomes after non-intubated versus intubated video-assisted thoracoscopic surgery for non-small cell lung cancer: a systematic review and meta-analysis
Source: Front Oncol. 2026 Aug 14;16:1933004. doi: 10.3389/fonc.2026.1933004 (PMC13521878; doi:10.3389/fonc.2026.1933004)
Supplement: Supplementary file 6 [file Table4.docx]

Supplementary Table S4. Newcastle-Ottawa Scale assessment for observational studies

| **Study** | **Selection** | **Comparability** | **Outcome** | **Total NOS score** | **Overall quality** |
| --- | --- | --- | --- | --- | --- |
| Chen 2011 | 4 | 1 | 1 | 6/9 | Moderate |
| Wu 2013 | 4 | 1 | 1 | 6/9 | Moderate |
| Liu 2016 | 4 | 2 | 1 | 7/9 | Moderate to high |
| AlGhamdi 2018 | 4 | 1 | 1 | 6/9 | Moderate |
| Furak 2020 | 4 | 1 | 2 | 7/9 | Moderate |
| Wang 2021 | 4 | 2 | 3 | 9/9 | High |
| Zheng 2021 | 4 | 2 | 3 | 9/9 | High |
| Chen 2022 | 4 | 2 | 2 | 8/9 | High |
| Wang 2022b | 4 | 2 | 2 | 8/9 | High |
| Wang 2022a | 4 | 2 | 1 | 7/9 | Moderate to high |
| Wu 2020 | 4 | 2 | 1 | 7/9 | Moderate to high |
| Zhao 2024 | 4 | 2 | 2 | 8/9 | High |
| Udelsman 2024 | 3 | 2 | 2 | 7/9 | Moderate to high |
| Zheng 2026 | 4 | 2 | 1 | 7/9 | Moderate to high |

**Note:** Observational studies were assessed using the Newcastle-Ottawa Scale. The selection domain is scored from 0 to 4, comparability from 0 to 2, and outcome assessment from 0 to 3, giving a maximum total score of 9. Higher scores indicate lower risk of bias. Overall quality was categorized pragmatically as high for scores of 7-9, moderate for scores of 5-6, and low for scores of 0-4. Final use indicates the main synthesis or narrative role for which the study contributed data in this review.
