# Supplementary material for: Long-term oncologic outcomes after non-intubated versus intubated video-assisted thoracoscopic surgery for non-small cell lung cancer: a systematic review and meta-analysis
Source: Front Oncol. 2026 Aug 14;16:1933004. doi: 10.3389/fonc.2026.1933004 (PMC13521878; doi:10.3389/fonc.2026.1933004)
Supplement: Supplementary file 7 [file Table5.docx]

Supplementary Table S5. GRADE certainty of evidence

| **Outcome** | **No. of studies/rows** | **Study design** | **Pooled effect** | **Heterogeneity** | **Risk of bias** | **Inconsistency** | **Imprecision** | **Indirectness** | **Publication bias** | **Certainty of evidence** | **Importance** | **Rationale** |
| --- | --- | --- | --- | --- | --- | --- | --- | --- | --- | --- | --- | --- |
| DFS/RFS | 5 studies | Mostly retrospective PSM/cohort evidence | HR 0.66 (0.50 to 0.87); *P*=0.003 | I^2^=0.0% | Serious | Not serious | Serious | Not serious | Undetected/very uncertain because k is small | Low | Critical | Downgraded for observational evidence base and imprecision/endpoint heterogeneity (DFS/RFS terminology); not downgraded for inconsistency. |
| OS | 6 studies | Mostly retrospective PSM/cohort evidence | HR 0.66 (0.48 to 0.91); *P*=0.010 | I^2^=0.0% | Serious | Not serious | Serious | Not serious | Undetected/very uncertain because k is small | Low | Critical | Downgraded for observational evidence base and imprecision; not downgraded for inconsistency because heterogeneity was negligible. |
| Total lymph nodes | 9 study rows | Mostly non-randomized comparative evidence | MD -0.30 (-1.91 to 1.31); *P*=0.715 | I^2^=63.3% | Serious | Serious | Serious | Not serious | Undetected/very uncertain because k is small | Low | Important | Downgraded for observational design; additional downgrading applied when heterogeneity or imprecision was substantial. |
| N1 lymph nodes | 4 study rows | Mostly non-randomized comparative evidence | MD -0.20 (-1.41 to 1.01); *P*=0.748 | I^2^=68.5% | Serious | Serious | Serious | Not serious | Undetected/very uncertain because k is small | Low | Important | Downgraded for observational design; additional downgrading applied when heterogeneity or imprecision was substantial. |
| N2 lymph nodes | 4 study rows | Mostly non-randomized comparative evidence | MD 0.06 (-1.53 to 1.65); *P*=0.940 | I^2^=51.8% | Serious | Serious | Serious | Not serious | Undetected/very uncertain because k is small | Low | Important | Downgraded for observational design; additional downgrading applied when heterogeneity or imprecision was substantial. |
| Total stations/groups | 3 study rows | Mostly non-randomized comparative evidence | MD 0.06 (-0.19 to 0.31); *P*=0.640 | I^2^=0.0% | Serious | Not serious | Serious | Not serious | Undetected/very uncertain because k is small | Very low | Important | Downgraded for observational design; additional downgrading applied when heterogeneity or imprecision was substantial. |
| N1 stations/groups | 2 study rows | Mostly non-randomized comparative evidence | MD -0.05 (-0.23 to 0.14); *P*=0.630 | I^2^=0.0% | Serious | Not serious | Serious | Not serious | Undetected/very uncertain because k is small | Very low | Important | Downgraded for observational design; additional downgrading applied when heterogeneity or imprecision was substantial. |
| N2 stations/groups | 2 study rows | Mostly non-randomized comparative evidence | MD -0.37 (-0.58 to -0.16); *P*=<0.001 | I^2^=0.0% | Serious | Not serious | Not serious | Not serious | Undetected/very uncertain because k is small | Very low | Important | Downgraded for observational design; additional downgrading applied when heterogeneity or imprecision was substantial. |
| Any recurrence | 3 studies | Mostly non-randomized comparative evidence | RR 0.68 (0.46 to 1.00); *P*=0.048 | I^2^=0.0% | Serious | Not serious | Serious | Not serious | Undetected/very uncertain because k is small | Low | Important | Downgraded for observational design and sparse events/small number of studies. |
| Death | 2 studies | Mostly non-randomized comparative evidence | RR 0.68 (0.27 to 1.70); *P*=0.407 | I^2^=26.4% | Serious | Not serious | Serious | Not serious | Undetected/very uncertain because k is small | Very low | Important | Downgraded for observational design and sparse events/small number of studies. |
| R0 resection | 2 studies | Mostly non-randomized comparative evidence | RR 0.98 (0.91 to 1.04); *P*=0.469 | I^2^=29.3% | Serious | Not serious | Serious | Not serious | Undetected/very uncertain because k is small | Very low | Important | Downgraded for observational design and sparse events/small number of studies. |

**Note:** Certainty of evidence was assessed at the outcome level using the GRADE framework. For observational evidence, certainty generally started as low and was further downgraded when there were serious concerns about risk of bias, inconsistency, indirectness, imprecision, or publication bias. HRs and RRs below 1 favor NIVATS for survival or adverse-event outcomes when the effect direction was harmonized; negative MDs indicate lower mean values in the NIVATS group. Publication-bias assessments were interpreted cautiously because most outcomes included fewer than 10 studies. Abbreviations: CI, confidence interval; DFS, disease-free survival; GRADE, Grading of Recommendations Assessment, Development and Evaluation; HR, hazard ratio; MD, mean difference; NIVATS, non-intubated video-assisted thoracoscopic surgery; OS, overall survival; RFS, recurrence-free survival; RR, risk ratio
